# Perfect collinearity not created equal: measuring and visualizing the severity of multi-collinearity of modern omics data

**DOI:** 10.1515/sagmb-2025-0043

**Published:** 2026-02-10

**Authors:** Wei Q. Deng, Radu V. Craiu, Lei Sun

**Affiliations:** Department of Psychiatry and Behavioural Neurosciences, 3710 McMaster University Peter Boris Centre for Addictions Research, St. Joseph’s Healthcare Hamilton, Hamilton, Canada; Department of Statistical Sciences, University of Toronto, Toronto, Canada; Department of Statistical Sciences, Dalla Lana School of Public Health, University of Toronto, Toronto, Canada

**Keywords:** genomic data, high-dimensional, multi-collinearity

## Abstract

Multi-collinearity frequently occurs in modern statistical applications and when ignored, can negatively impact model selection and statistical inference. Though perfect collinearity is always present in “*n* < *p*” data, we demonstrate that perfect collinearity arises differently, from diverse data redundancy patterns and/or data dimensions. Classic tools and measures that were developed for “*n* > *p*” data cannot be used to distinguish or visualize these patterns in the high-dimensional regime. Here we propose 1) new individualized measures that can be used to visualize patterns of perfect collinearity, and subsequently 2) global measures to assess the overall burden of multi-collinearity irrespective of data dimensions. We applied these measures to the human X chromosome data to understand similarity and differences in linkage disequilibrium structure due to sex and genetic features. The measures can highlight gene regions of excessive multi-collinearity and contrast the severity of perfect collinearity between different sexes. Utility of these measures to high-dimensional statistical application were also discussed.

## 1 Introduction

In the current age of information, statisticians often benefit from the ubiquitous capacity to measure multiple features or variables for each sample or observation. For example, modern genomic technology generated tens of millions of genetic features for hundreds of thousands of samples in the UK Biobank (Sudlow et al. 2015), and proteomic assay can characterize thousands of circulating proteins for hundreds of individuals (Suhre et al. 2021). A somewhat reversal of this fortune occurs when the number of samples *n* does not keep pace with the number of features *p*, thus leading to the high-dimensional data matrices *X* ∈ *R* ^*n*×*p*^ for which *n* < *p* (Donoho et al. 2000). For such data, perfect collinearity, in which case one feature vector has an exact linear relationship with the remaining ones, is inevitable and produces damaging effects on model selection and statistical inference (Fan and Lv 2008). However, not all perfect collinearity scenarios are created equal. In some cases, perfect collinearity is the sole result of data dimensions and can be resolved by sampling additional observations such that *n* > *p*. While in other cases, the cause of perfect collinearity is deep-rooted in the data generative process whereby the sample covariance matrix could be better approximated by a low-rank solution. Consequently, assessing the severity of perfect collinearity in high dimensions is not straightforward as such a measure must factor in both the number of variables involved relative to the sample size as well as the degree of collinearity among arbitrary subsets of the variables. The absence of a severity measure for perfect collinearity in high-dimensional settings seems disconnected from the fact that variable selection remains pivotal in balancing model accuracy and interpretability (George 2000, Wasserman and Roeder 2009). Moreover, identifying which variables are collinear or even redundant can be just as important as finding a subset with high explanatory power.

There are some potential candidates for measuring multi-collinearity in high dimensions. The *Red* indicator (Kovács et al. 2005) has been proposed to quantify the average level of correlation in the data. An almost identical quantity is the root mean square correlation over all *p*(*p* − 1)/2 pairs variables introduced in Efron (2010), also a key component in the approximation of covariance. These are single number measures that do not point to any specific variables, but cast light on the appropriate next-steps. For example, they can guide the implementation of regularization or penalization techniques in the context of high-dimensional linear regression, such as least absolute shrinkage and selection operator (lasso; Santosa and Symes 1986, Tibshirani 1996) or elastic net regularization (Zou and Hastie 2005).

We can perhaps learn from the more accessible scenario of *n* > *p*, where diagnostic measures to assess severity of multi-collinearity have been reliably used, especially in the context of linear regression (Belsley 2014, Farrar and Glauber 1967, Marquaridt 1970). These measures fall into two categories, one relying on a collection of numbers measuring the impact or burden of multi-collinearity on each individual variable, and the other category that uses a single number to summarize the severity of multi-collinearity of all variables or a subset of the variables.

Examples of the former include a class of measures that incorporate various functions of the estimated coefficient of determination $R_{j}^{2}$ from linear regression models. In essence, this type of measure leverages information on how well the *j*th variable is explained by linear combinations of the others as an indicator of the severity of collinearity. Among them, the most commonly used is the variance inflation factor (*VIF*; Marquaridt 1970), defined by

$${VIF}_{j}=\frac{1}{1-R_{j}^{2}},$$

intuitively interpreted as the inflating factor for the variance of the estimated regression coefficient for the *j*the variable. *VIF* not only captures the degree of multi-collinearity for each variable, but also illustrates a direct impact on inference in linear regression models (Fox 1984). Departing from examining *X* alone for multi-collinearity, a corrected *VIF* (denoted CVIF; Curto and Pinto 2011) was proposed to differentiate variables based on whether the redundant information is predictive of the response variable or not. The corrected *CVIF* is preferred over *VIF* when the redundancy among variables is unrelated to the response variable (Curto and Pinto 2011). These individual-valued measures offer a mechanism to remove variables implicated in near or perfect multi-collinearity according to a pre-defined threshold (e.g. *VIF*_*j*_ or *CVIF*_*j*_ > 10), and thus ensure coefficient estimates of the remaining variables using ordinary least square (OLS) are numerically stable.

The second class of measures uses a single number to summarize multi-collinearity. The most notable being the condition number, defined by the ratio of the largest and smallest singular values of a data matrix (Geurts 1982, Rice 1966). It is directly related to the matrix solution of a linear system and describes the degree to which the matrix *X* ^*T*^ *X* is ill-conditioned. For a scaled data matrix with unit variance in each column, a value between 15 and 30 is considered moderately problematic and severe if above 100 (Belsley et al. 2005). Closely related is the condition index (Belsley 2014), which is defined by the square root of ratio of the largest eigenvalue and each of the remaining eigenvalue of *X* ^*T*^ *X*. The number of condition indices above a threshold further indicates the number of near or perfect multi-collinear relationships in the data. Other global measures include those examining the determinant of *X* ^*T*^ *X*, such as the Farrar-Glauber test statistic (Farrar and Glauber 1967) that evaluates a function of the determinant of *X* ^*T*^ *X*.

In practice, application of the two classes of measures need not be mutually exclusive. In fact, it has been shown that *VIF*s are bounded above by the squared condition number (Berk 1977, Salmerón et al. 2018), implying that there could be additional information in condition number that is not captured by *VIF*s. Indeed, sometimes problematic variables are restricted to a particular subset while their individual *VIF*s might not all be strong enough to be picked up at the recommended threshold. A generalization of the *VIF* has been proposed by Fox and Monette (1992) to measure an arbitrary subset of variables for evidence of multi-collinearity, which can be used to identify specific sources of imprecision.

Under *n* < *p*, measures such as *VIF* cannot be reliably calculated, while overall measures that rely on the sample eigenvalues or singular values could be misleading, as we demonstrate in Section 3.2. Further, the usual approach to visualize pairwise relationship quickly becomes cumbersome as the number of combinations increases exponentially. Finally, though the *Red* indicator or the root mean square correlation can be useful as an overall summary, they do not fully address the complexity of multi-collinearity in high-dimensional settings.

This paper contributes in two new ways to the study of multi-collinearity in the case of high-dimensional data. First, it introduces new measures for the severity of multi-collinearity derived via the singular value decomposition (SVD) of *X*. Second, it uses these novel measures to establish whether the multi-collinearity is due to all or just a few of the variables. The remaining paper is organized as follows. Section 2 introduces the individual-valued measures, presents their empirical properties, and motivates an overall summary measure. Section 3 illustrates the utility of these measures to visualize and characterize multi-collinearity, making them an attractive option for exploratory data analysis on high-dimensional data. Section 4 demonstrates their application to genotype data from the 1000 Genomes Project (1000 Genomes Project Consortium and others 2015) to learn about the different patterns of multi-collinearity in genetic variations arising from diverse ancestral backgrounds.

## 2 A severity measure of multi-collinearity

Let $X\in R^{n\times p}$ be the observed data matrix with each column standardized to have sample mean 0 and variance 1. We are interested in the high-dimensional data setting (*n* < *p*) that is the signature of large-scale data such as those arising from genomic applications, but results also naturally generalize to the data rich setting (*n* > *p*). Denote the SVD of *X* by *UDV* ^*T*^, where columns of $U\in R^{n\times \left(n-1\right)}$ are the left singular vectors, *D* is a diagonal matrix with singular values *d*_1_ ≥ *d*_2_ ≥⋯ ≥ *d*_*n*−1_ ≥ 0, and columns of $V\in R^{p\times \left(n-1\right)}$ are the right singular vectors. The column standardization results in the loss of one degree of freedom such that $\sum_{i^{\prime }=1}^{n-1}d_{i^{\prime }}^{2}=\left(n-1\right)p$ , which is the sum of the main diagonal elements of $X^{T}X\in R^{p\times p}$ . Notice that by permitting *d*_*i*′_ = 0, the matrix *X* is allowed to be rank deficient, which would be the consequence of perfect collinearity involving two or more variables.

Define the *right severity* measure of multi-collinearity by

$$SR_{j}=V_{j.}D^{4}V_{j.}^{T}=\overset{n-1}{\underset{i^{\prime }=1}{\sum }}v_{ji^{\prime }}^{2}d_{i^{\prime }}^{4},$$

where *V*_*j*._ denotes the *j*th row and *v*_*ji*′_ the (*j*, *i*′)th entry of *V*.

Naturally, the duality of SVD allows the definition a *left severity*:

$$SL_{i}=U_{i.}D^{4}U_{i.}^{T}=\overset{n-1}{\underset{i^{\prime }=1}{\sum }}u_{ii^{\prime }}^{2}d_{i^{\prime }}^{4},$$

where *U*_*i*._ denotes the *i*th row and *u*_*ii*′_ the (*i*, *i*′)th entry of *U*.

Notice that these two measures are equal when *X* is symmetric (i.e. *X* = *X* ^*T*^). Both *SR* and *SL* leverage the spectrum of singular values of *X*, similar to other measures of multi-collinearity, but also the singular vectors, which are used to assign a value to each variable/sample through the weighted *l*_2_ norm of the corresponding right/left singular vector. In this construction, the singular values comprehensively capture the variance spectrum, and weighting by their respective singular vectors creates individualized measures irrespective of the data dimensions. Since the top singular values bear the higher burden of capturing the variance in *X* ^*T*^ *X* and contribute more weight to the measures, *SL*_*i*_ and *SR*_*j*_ are termed the univariate burden of variance adjustment (uBVA) measure for left and right severity, respectively.

### 2.1 Basic properties

Without invoking any distributional or data dimensions assumptions, we first establish three basic properties of ${\left\{SR_{j}\right\}}_{j=1,\ldots ,p}$ given any observed $X\in R^{n\times p}$ with each column standardized to have sample mean 0 and variance 1.

**Property 2.1.**

(2.1) $$\overset{p}{\underset{j=1}{\sum }}SR_{j}=\overset{n}{\underset{i=1}{\sum }}{SL}_{i}=\overset{n-1}{\underset{i^{\prime }=1}{\sum }}d_{i^{\prime }}^{4}$$

**Remark 2.1.**

Though the sums of *SR*_*j*_ and *SL*_*i*_ are the same, the collective pattern of these values is influenced by the underlying column and row dependence, respectively.

**Property 2.2.**

(2.2) $$SR_{j}=\overset{n-1}{\underset{i^{\prime }=1}{\sum }}v_{ji^{\prime }}^{2}d_{i^{\prime }}^{4}={\left(n-1\right)}^{2}\overset{p}{\underset{j^{\prime }=1}{\sum }}r_{jj^{\prime }}^{2},$$

where *r*_*jj*′_ denotes the sample Pearson’s correlation coefficient between the *j*th and *j*′th columns. Note that since the data had been column-standardized, we have $r_{jj}^{2}=1$ for all *j* = 1, *…*, *p*.

**Remark 2.2.**

The equivalent expression of *SR*_*j*_, shown in Equation (2.2), offers some intuition to the construction of the measure. The magnitude of *SR*_*j*_ scales with the variance of the *j*th column itself as well as any redundancy due to its correlation with all other columns. The larger *SR*_*j*_ is, the more the *j*th column is involved in multi-collinearity, quantified by the number and severity of these collinear relationships.

**Remark 2.3.**

Since $r_{jj^{\prime }}^{2}\in \left[0,1\right]$ , the maximum value of *SR*_*j*_ is bounded by (*n* − 1)^2^ *p*, while the minimum possible value is bounded by (*n* − 1)^2^. These bounds apply to any $X\in R^{n\times p}$ , irrespective of *n* > *p* or *n* < *p*. However, a tighter bound is established in the next property when we restrict data dimensions to be *n* < *p*.

**Property 2.3.** When *n* < *p*,

$$SR_{j}\in \left[\frac{{\left(n-1\right)}^{2}}{\sum_{i^{\prime }=1}^{n-1}v_{ji^{\prime }}^{2}},d_{1}^{2}\left(n-1\right)\right].$$

**Remark 2.4.**

When *n* > *p*, the lower bound becomes (*n* − 1)^2^ assuming all *p* columns are mutually orthogonal. However, when *n* < *p*, *X* has at most min(*n*, *p*) − 1 orthogonal columns; the restriction of dimension (*n* < *p*) leads to a tighter lower bound than (*n* − 1)^2^ because the squared row norm of a column orthogonal matrix is strictly less than 1 (i.e. $\sum_{i^{\prime }=1}^{n-1}v_{ji^{\prime }}^{2}<1$ ). In fact, following from $\sum_{j=1}^{p}\sum_{i^{\prime }=1}^{n-1}v_{ji^{\prime }}^{2}=n-1$ , the lower bound $\frac{{\left(n-1\right)}^{2}}{\sum_{i^{\prime }=1}^{n-1}v_{ji^{\prime }}^{2}}$ is expected to vary for each *j*, but the smallest such lower bound is strictly smaller than (*n* − 1)*p*. In other words, the smallest value *SR*_*j*_ can take under a high-dimensional data setting is greater than that under the one with *n* > *p*, the result of spurious correlation as discussed in Fan et al. (2012). Clearly, the severity increases with an increasing *p*/*n* ratio. Meanwhile, as $\sum_{i^{\prime }=1}^{n-1}d_{i^{\prime }}^{2}=\left(n-1\right)p$ , the upper bound $d_{1}^{2}\left(n-1\right)$ is also bounded above by the naive upper bound of (*n* − 1)^2^ *p*, but these two are equivalent when columns of *X* are identical and $\sum_{i^{\prime }=1}^{n-1}d_{i^{\prime }}^{2}=d_{1}^{2}$ .

**Remark 2.5.**

Under column standardization, the bounds of *SL*_*i*_ are not directly informative as the singular values are scaled to have unit column variance. Thus, we provide bounds for *SL*_*i*_ assuming row standardization and *n* < *p*, which implies that $\sum_{i^{\prime }=1}^{n-1}d_{i^{\prime }}^{2}=n\left(p-1\right)$ and $\sum_{i^{\prime }=1}^{n-1}u_{ii^{\prime }}^{2}d_{i^{\prime }}^{2}=p-1$ . The upper bound is then:

$${SL}_{i}=\overset{n-1}{\underset{i^{\prime }=1}{\sum }}u_{ii^{\prime }}^{2}d_{i^{\prime }}^{4}\leq d_{1}^{2}\overset{n-1}{\underset{i^{\prime }=1}{\sum }}u_{ii^{\prime }}^{2}d_{i^{\prime }}^{2}\leq d_{1}^{2}\left(p-1\right),$$

and the lower bound follows from the Cauchy-Schwarz inequality:

$${SL}_{i}=\overset{n-1}{\underset{i^{\prime }=1}{\sum }}u_{ii^{\prime }}^{2}d_{i^{\prime }}^{4}\geq \left(\overset{n-1}{\underset{i^{\prime }=1}{\sum }}u_{ii^{\prime }}^{2}\right){\left(\overset{n-1}{\underset{i^{\prime }=1}{\sum }}u_{ii^{\prime }}^{2}d_{i^{\prime }}^{2}\right)}^{2}\geq {\left(p-1\right)}^{2}.$$

Thus far, we have not invoked any distributional assumptions. By assuming each row of *X* follows a multivariate normal distribution, the expected value of *SR*_*j*_ can be expressed in terms of the true covariance matrix and data dimensions when *n* > *p* − 1.

**Lemma 2.1.**

Suppose **rows** of $X\in R^{n\times p}$ are independent and identically distributed (i.i.d) normal random vectors, i.e. for *i* ∈ {1, *…*, *n*}, $x_{i}∼N\left(0,\Sigma \right)$ , where *Σ* is positive definite and *Σ*_*j*_ is the *j* column of *Σ*, then

$$\text{E}\left(SR_{j}\right)=\left(n-1\right)\Sigma_{jj}\text{tr}\left(\Sigma \right)+n\left(n-1\right)\Sigma_{j}^{T}\Sigma_{j}.$$

Without loss of generality, assume *Σ*_*jj*_ = 1, the expectation can be simplified to

(2.3) $$\text{E}\left(SR_{j}\right)=\left(n-1\right)p+n\left(n-1\right)\Sigma_{j}^{T}\Sigma_{j},$$

which reveals the direct impact of data dimension, *p*, on the severity of multi-collinearity.

This result suggests that the expected value of the proposed measure *SR*_*j*_ has two components, one that is driven by data dimensions (*n* and *p*) and the other by the non-zero off-diagonal entries in the corresponding columns of Σ.

**Remark 2.6.**

The above result does not apply to the *n* < *p* setting as the scaled sample covariance *X* ^*T*^ *X* no longer follows a Wishart distribution due to the insufficient degrees of freedom (Wishart 1928). In this case, *X* ^*T*^ *X* is said to have a singular Wishart distribution and explicit moments are not available (Srivastava 2003). An alternative solution is to consider a low-rank approximation of *X* ^*T*^ *X* with rank *r* (*r* < *n*) and compute the approximated expectation, at the cost of slightly underestimating E(*SR*_*j*_).

Though the main focus here was on the empirical properties of these measures without distributional assumptions, it is possible to further characterize the statistical properties of *SR*_*j*_ or *SL*_*i*_ according to behaviours of the singular values and vectors using random matrix theory such as in Bai (2008). This will be the subject of future work.

Following property 2.2, it is natural to define a scaled measure:

(2.4) $$sR_{j}=\frac{SR_{j}}{{\left(n-1\right)}^{2}}=\overset{p}{\underset{j^{\prime }=1}{\sum }}r_{jj^{\prime }}^{2}\in \left[1,p\right],$$

as it has a more natural interpretation of being the sum of squared pairwise Pearson’s correlation coefficients. From the row perspective, *sL*_*i*_ can also be defined similarly, provided the data had been row standardized:

(2.5) $$sL_{i}=\frac{{SL}_{i}}{{\left(p-1\right)}^{2}}\in \left[1,n\right].$$

As the results in Section 2.1 can be conveniently expressed by a rescaling, the bounds on *sR*_*j*_ become:

(2.6) $$sR_{j}\in \left[\frac{1}{\sum_{i^{\prime }=1}^{n-1}v_{ji^{\prime }}^{2}},\frac{d_{1}^{2}}{n-1}\right],$$

where $\frac{1}{\sum_{i^{\prime }=1}^{n-1}v_{ji^{\prime }}^{2}}\leq 1$ , taking equality when *n* > *p*; and $\frac{d_{1}^{2}}{n-1}\leq p$ , taking equality when all columns are identical (i.e. $\sum_{i^{\prime }=1}^{n-1}d_{i^{\prime }}^{2}=d_{1}^{2}=\left(n-1\right)p$ ). The upper and lower bounds are expected to be numerically close when multi-collinearity is driven by spurious correlation due to *n* < *p* alone, but further apart as both the number and strength of multi-collinear relationships increase.

An informative interpretation of *sR*_*j*_ in high-dimensional settings is through its inverse, 1/*sR*_*j*_, which can be viewed as a proxy for the effective degrees of freedom per variable. When multicollinearity arises purely due to high dimensionality (e.g., *p* ≫ *n*), *sR*_*j*_ scales approximately with *p*/*n*, and the average of 1/*sR*_*j*_ across variables reflects the effective sample size per parameter, see Supplementary Section 6. A heuristic threshold of $\sum_{j=1}^{p}1/sR_{j}\geq 0.3n$ aligns with effective sample size criteria used in high-dimensional Bayesian modeling, where stable estimation typically requires at least 20–30 % of a sample per parameter (Morita et al. 2008, Piironen and Vehtari 2017). This suggests that multicollinearity is driven primarily by dimensionality rather than by structured dependence. Conversely, values exceeding this threshold may reflect true correlation structure in the data-generating process. *The result in Lemma 2.1 becomes:*

(2.7) $$\text{E}\left(sR_{j}\right)=\frac{p}{n-1}+\frac{n}{n-1}\Sigma_{j}^{T}\Sigma_{j},$$

which reveals the direct impact of relative data dimensions, *p*/(*n* − 1), on the severity of multi-collinearity.

### 2.2 sRs: a unifying measure of multi-collinearity

Since {*sR*_*j*_} is considered the individualized measure of multi-collinearity, we propose a summary measure *sRs* as a weighted sum of *sR*_*j*_ with two components:

(2.8) $$sRs=\frac{\sum_{j=1}^{p}sR_{j}-p}{p\left(p-1\right)}\times \frac{w_{1}+w_{2}}{2}+\frac{\sum_{j=1}^{p}sR_{j}-p}{p\left[d_{1}^{2}{\left(n-1\right)}^{-1}-1\right]}\times \left(1-\frac{w_{1}+w_{2}}{2}\right)\in \left[0,1\right],$$

where

$$w_{1}=\frac{\sum_{d_{i}^{2}>p}d_{i}^{2}}{\sum \overset{2}{\underset{i}{d}}}$$

adjusts the weight of “bulk” behaviour more heavily when *n* < *p* and

$$w_{2}=\frac{\sum_{d_{i}^{2}>{\left(\sqrt{n}-\sqrt{p}\right)}^{2}}d_{i}^{-2}}{\sum \overset{-2}{\underset{i}{d}}},$$

so that 1 − *w*_2_ adjusts the weight of “local” behaviour more pronouncedly when *n* > *p*.

We refer to the first component in (2.8) as bulk sRs (*BsRs*):

(2.9) $$BsRs=\frac{\sum_{j=1}^{p}sR_{j}-p}{p\left(p-1\right)},$$

which captures the overall burden of multi-collinearity, weighted by the proportion of singular values exceeding their averaged value (maximum of *n* or *p*). The second component in (2.8) is designed specifically to account for the number of “locally” strong relationships and defined as local sRs (*LsRs*):

(2.10) $$LsRs=\frac{\sum_{j=1}^{p}sR_{j}-p}{p\left[d_{1}^{2}{\left(n-1\right)}^{-1}-1\right]}.$$

The proposed global measure sRs is designed to capture both bulk and local aspects of multi-collinearity. The decomposition is motivated by the observation that overall burden or bulk signal is driven by the concentration of variance in a few dominant singular values (i.e., a low-rank approximation of the data), while local collinearity arises when individual variables are nearly linear combinations of small subsets of others, which can occur even when the singular spectrum decays gradually. The *sRs* “bulk” component is mathematically equivalent to the squared *Red*, defined as

(2.11) $$\text{Red}=\sqrt{\frac{\text{tr}\left[X^{T}XX^{T}X-{\left(n-1\right)}^{2}I_{p}\right]}{p\left(p-1\right){\left(n-1\right)}^{2}}},$$

and both describe the “average correlation” of all variables. It is sensitive to spectral dominance, and reaches its maximum when a single singular value dominates the spectrum. But the addition of a “local component” in *sRs* helps account for strong “local” collinearity that involves only a subset of the variables. The denominator in Equation (2.10) rescales the total excess in *sR*_*j*_ values by the theoretical maximum that could arise if all variables were perfectly aligned with the top singular vector, representing a scenario of extreme local collinearity.

While *BsRs* is normalized by *p*(*p* − 1), which corresponds to the maximum number of possible pairwise correlations that equal to 1, the local component *LsRs* is normalized by $p\left[\frac{d_{1}^{2}}{n-1}-1\right]$ , which reflects the maximal variable-specific collinearity under the extreme case where all columns align with the top singular vector, see Equation (2.6). This ensures that *BsRs* captures diffuse global collinearity, while *LsRs* is tuned to detect sharp local alignment that might not influence global averages. Notice that, *p* is used as the upper bound for *sR*_*j*_ when the “bulk” behaviour dominates, i.e. the majority of variance is in the leading singular values, while the maximum given by (2.6) is used when the top singular values do not dominate others. In other words, the combined measure includes each *sR*_*j*_ but weighs the signal relatively.

The weights *w*_1_ and *w*_2_ were chosen to balance the contribution of the two components according to the shape of the singular value spectrum. Specifically, *w*_1_ emphasizes bulk behaviour by capturing the proportion of variance explained by singular values greater than *p* (or *n*), while *w*_2_ downweights local contributions when the spectrum is sharply peaked. When the spectrum decays smoothly, the local component contributes more, as each variable projects meaningfully onto multiple singular values. Thus, while *BsRs* increases when variance is compressed into a few directions, *LsRs* is maximized when redundant variance is more evenly distributed across a subset of variables. The lower bound of *sRs* = 0 is achieved when *n* > *p* and columns of *X* are mutually orthogonal; the upper bound of *sRs* = 1 is achieved when columns of *X* are identical. Our proposed *sRs*, along with *LsRs* and *BsRs*, have better interpretation as compared to the *Red* indicator, e.g. a value closer to 0 suggests no evidence of multi-collinearity. In contrast, a value closer to 1 indicates severe multi-collinearity due to 1) a subset of variables (local), a scenario that *Red* was unable to capture; 2) a large number of variables (bulk); 3) both 1) and 2). The relative contribution of *LsRs* and *BsRs* to *sRs* can be used to suggest which one of the scenarios constitutes the main driver of observed multi-collinearity.

## 3 On the use of right severity measure for data exploratory analysis

This section focuses on the utility of *sR*_*j*_ and *sRs* (*LsRs* and *BsRs*) through simulation studies. In Section 3.1, we applied *sR*_*j*_ to high-dimensional data simulated under different covariance structures to confirm basic properties of *sR*_*j*_ and to explore its use for initial data analysis. In Section 3.2, we compared *sRs* (*LsRs* and *BsRs*) with existing measures to assess multi-collinearity in data generated under various multi-collinearity patterns assuming either a high-dimensional (*n* < *p*) or data rich scenario (*n* > *p*).

### 3.1 Visualizing data covariance structure

As each *sR*_*j*_ is a weighted sum of singular values and that the spectrum of singular values is driven by the covariance structure from which the data were sampled, it is tempting to use ${\left\{sR_{j}\right\}}_{j=1,\ldots ,p}$ to identify certain “signatures” in the sample covariance through a visual inspection. Property 2.2 (i.e. $sR_{j}=\sum_{j^{\prime }=1}^{p}r_{jj^{\prime }}^{2}$ ), suggests that the observed range of ${\left\{sR_{j}\right\}}_{j=1,\ldots ,p}$ is directly related to the number and strength of squared pairwise correlation coefficients. While Property 2.3 implies that the observed extremes of ${\left\{sR_{j}\right\}}_{j=1,\ldots ,p}$ are specific to the data beyond dimensions. In summary, the observed patterns of *sR*_*j*_ are reflective of the singular values and can be visualized to give a fuller picture of the covariance structure.

With simulated examples, we demonstrate the usefulness of this measure to differentiate some representative covariance structures. The data dimensions were fixed at *n* = 500 and *p* = 1, 000. Following the standard notation, we use *J*_*p*_ to denote an *p* × *p* matrix of ones and *I*_*p*_ a *p* × *p* identity matrix. Each row of *X* was generated according to $x_{i}∼N\left(0,\Sigma \right)$ , where

- *Σ* = *I*_*p*_ denotes a case of identity covariance,
- *Σ* = *J*_*p*_ *ρ* + (1 − *ρ*)*I*_*p*_, a compound symmetric structure with *ρ* = 0.2,
- *Σ*_*i*,*j*_ = *ρ* ^|*j*−*i*|^, a first order autoregressive (AR1) structure with *ρ* = 0.8.
- *Σ* = diag[(0.1 × *J*_*p*/2_ + 0.9*I*_*p*/2_), (0.4 × *J*_*p*/4_ + 0.6*I*_*p*/4_), (0.6 × 1_*p*/4_ + 0.4*I*_*p*/4_)], a covariance with three compound symmetric blocks,
- *Σ* = *LL* ^*T*^ + *ζ* ^2^ *I*_*p*_, a spiked covariance with two distinct eigenvalues; the low-rank representation *L* = *V*_,1:*k*_ *O* is given by the first *k* columns of the right singular vectors, *k* = 10, $O=\frac{1}{\sqrt{n}}\mathrm{diag}\left[\sqrt{10},\ldots ,\sqrt{10}\right]$ , and *ζ* ^2^ = 0.4,
- *Σ* = *LL* ^*T*^ + *ζ* ^2^ *I*_*p*_, a spiked covariance with *k* + 1 distinct eigenvalues; *k* = 10, *O* = [*o*_1_, *…*, *o*_*k*_], where $o_{k}^{2}=2+\zeta^{2}$ and *ζ* ^2^ = 0.4.

For the spiked covariance models, when *O* is not given explicitly, we assumed $o_{1}^{2},\ldots ,o_{k-1}^{2}$ follow an exponential decay, which uniquely determined the values via the constraints imposed by $o_{k}^{2}=1+\zeta^{2}$ and $\sum_{i=1}^{k}o_{i}^{2}+p\zeta^{2}=p$ .

**Figure 1:**
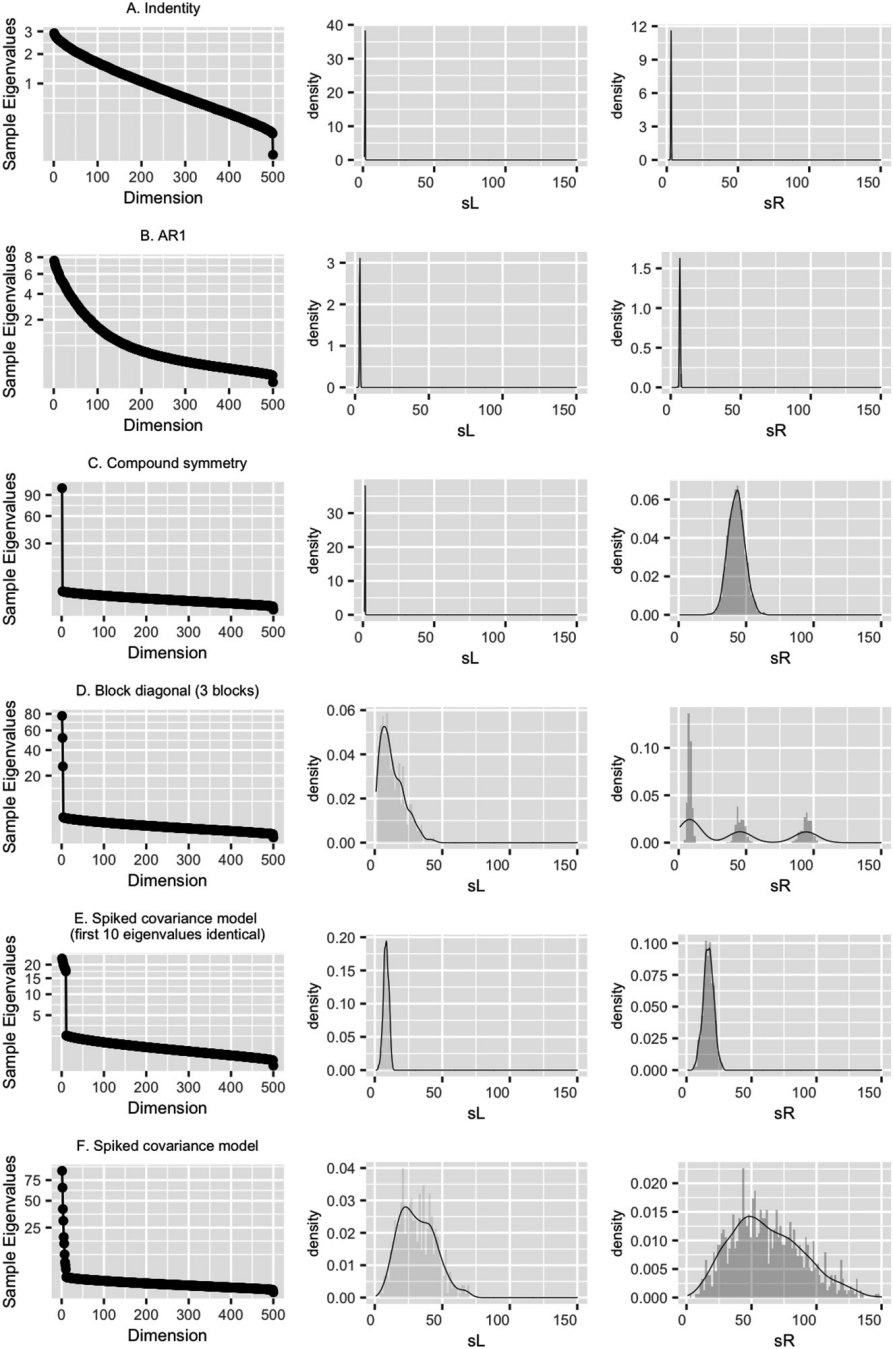
Empirical distributions of sample eigenvalues, *sL*_*i*_ and *sR*_*j*_ under different data covariance structures.

The sample eigenvalues (or normalized squared singular values) shown in Figure 1(A–F) have distinct patterns under each structure: a relatively smooth decay in the case of an identity covariance (A) and AR1 structure (C); a sharp drop is identified for the compound symmetry case (B), the block-wise compound symmetric covariance (D), and the spiked covariance with two identical true eigenvalues (E); and finally a visible “elbow” for the spiked covariance with *k* + 1 unique true eigenvalues (F). However, these might not be sufficient to differentiate the block-diagonal and the low-rank spiked covariance cases. This is where {*sR*_*j*_} could lend additional information.

The identity case (Figure 1A) is equivalent to each entry having a standard normal distribution and Marchenko-Pastur law (Marchenko and Pastur 1967) applies. We expected the observed *sR*_*j*_ and *sL*_*i*_ to fall between $\left(\frac{p}{n},{\left(1+\sqrt{n/p}\right)}^{2}\frac{p}{n}\right)$ and $\left(1,\frac{d_{1}^{2}}{p}\right)$ , which translate to (2.00, 5.83) and (1.00, 2.91), respectively. These are consistent with the observed ranges of (2.72, 3.31) for *sR*_*j*_ and (1.15, 1.81) for *sL*_*i*_ (Figure 1A). The observed *sR*_*j*_ had a tight symmetrical shape, with most values centred around its observed median (3.005), which was approximately the same as its observed mean (3.007).

The sample eigenvalues of an AR1 structure (Figure 1C) behaved similarly to that of an identity covariance with the additional variance for each principal direction contributed by only nearby variables. The empirical pattern should also be unimodal and symmetric around its mean/median, but differ in the extremes from the identity case. In practice, for large *p*, the majority of *sR*_*j*_ should have expected values close or equal to the maximum because *ρ* ^|*j*−*i*|^ diminishes quickly as |*j* − *i*| increases. For fixed data dimensions, the observed range of (3.65, 5.70) was attributed to the parameter value *ρ* = 0.3. As *ρ* increases, the range will become wider with a smaller minimum and a larger maximum.

When the true covariance matrix *Σ* = (1 − *ρ*)*I*_*p*_ + 1_*p*_ *ρ* has a compound symmetric structure (Figure 1B), the largest population singular value is $\sqrt{n\left[1+\left(p-1\right)\rho \right]}$ and the remaining *n* − 1 singular values are $\sqrt{\left(1-\rho \right)\left(p-1\right)}$ . For small *ρ* values, *sR*_*j*_ is influenced mostly by the top singular values and their corresponding singular vectors. Since all pairwise variables have the same 2 × 2 covariance, as expected, the empirical distribution of *sR*_*j*_ was roughly symmetric with a unimodal shape that peaked around the mean (42.84) and median (42.81).

When *Σ* exhibits a block structure and each block is compound symmetric:

$$\Sigma =\left[\begin{array}{ccc} \Sigma_{1} & 0 & 0\\ 0 & \Sigma_{2} & 0\\ 0 & 0 & \Sigma_{3} \end{array}\right],$$

the empirical patterns of ${\left\{sR_{j}\right\}}_{j}$ should feature three visible modes corresponding to each block similarly described for a compound symmetric structure (Figure 1D). However, note that if any two blocks are identical, *sR*_*j*_ would simply be duplicated for the identically distributed variables in these two blocks. As a result, two of the three modes would completely overlap, forming a single mode. In general, the number of modes corresponding to the number of unique blocks while the within block pattern depends on the structure of that block.

The last scenario focused on variables with varying magnitudes of pairwise correlation such that the true covariance followed a spiked structure whereby *Σ* = *VO* ^2^ *V* ^*T*^ + *ζ* ^2^ *I*_*p*_ (Figure 1E and F). Though challenging to estimate sample covariance directly, the empirical patterns of *sR*_*j*_ was mostly be driven by the top singular values whose true values are proportional to diagonal elements of *V*. As a result, the empirical patterns spread much wider (ranging from 8.13 to 138.43) and no modes would be unambiguously identified unless the top singular values were truly identical. Indeed, when the true covariance has equal eigenvalues, the observed {*sR*_*j*_} (ranging from 5.76 to 30.26) can be made to resemble a compound symmetry covariance by varying the two unique eigenvalues. Nevertheless, it can be argued that the compound symmetry covariance is actually a special case of a spiked covariance model with only one spike.

### 3.2 Measuring the severity of multi-collinearity

We have proposed {*sR*_*j*_} and *sRs* as individual-valued and summary-level measures, respectively, to assess severity of multi-collinearity in high dimensions where existing measures fall short. Here we benchmark their performance against alternatives under high-dimensional settings (*n* < *p*) and data rich settings (*n* > *p*). Sample size of the simulated data was varied (*n* = 100 and *n* = 500), and the number of variables was fixed at *p* = 1,000 for the high-dimensional or *p* = 50 for the data rich scenarios.

In contrast to the previous simulation study of general covariance structures, we specified covariance matrix to represent no multi-collinearity via an identity matrix (orthogonal design), multi-collinearity through two near-collinear variables (collinear), a moderate level of multi-collinearity impacting all variables through a compound-symmetric covariance matrix (CS), a severe multi-collinearity impacting all variables through a spiked covariance model (spiked covariance), and the most severe case of nearly all variables are identical (almost perfect multi-collinear). To make a more interesting comparison, we also included a block-wise scenario where two variables are near-collinear but the remaining variables follow a compound-symmetric covariance structure. Similar to the simulations in Section 3.1, each row of *X* was generated according to $x_{i}∼N\left(0,\Sigma \right)$ , where

*Σ* = *I*_*p*_,
(local) $\Sigma =\mathrm{diag}\left[0.99\times 1_{2}+\sqrt{1-0.99^{2}}I_{2},I_{p-2}\right]$ ,
(bulk) *Σ* = *ρ*1_*p*_ + (1 − *ρ*)*I*_*p*_ with *ρ* = 0.3 or *ρ* = 0.99,
(bulk and local) $\Sigma =\mathrm{diag}\left[0.99\times 1_{2}+\sqrt{1-0.99^{2}}I_{2},\rho 1_{p-2}+\left(1-\rho \right)I_{p-2}\right]$ with *ρ* = 0.3,
(local) *Σ* = *LL* ^*T*^ + *ζ* ^2^ *I*_*p*_ with *k* = 10, $o_{k}^{2}=1+\zeta^{2}$ , and *ζ* ^2^ = 0.4.

The alternative measures include *VIF*, the condition number, and the *Red* indicator. As *VIF* can only be sensibly applied when *n* > *p*, it was only included for comparisons in the data rich scenarios. The condition number is defined by the ratio of the largest and smallest singular values of a data matrix and describes the degree to which the matrix *X* ^*T*^ *X* is ill-conditioned. For the high-dimensional case, it was taken to be $\frac{d_{1}}{d_{n-1}}$ due to the column standardization; while for the data rich case, it was calculated as $\frac{d_{1}}{d_{p}}$ . By design, the condition number captures the degree of multi-collinearity rather than the number of collinear relationships. In other words, it evaluates the worst case scenario and as a result, one perfect collinear relationship is all it takes to reach infinity, i.e. when *d*_*p*_ or *d*_*n*−1_ is exactly zero.

#### 3.2.1 High-dimensional settings

**Figure 2:**
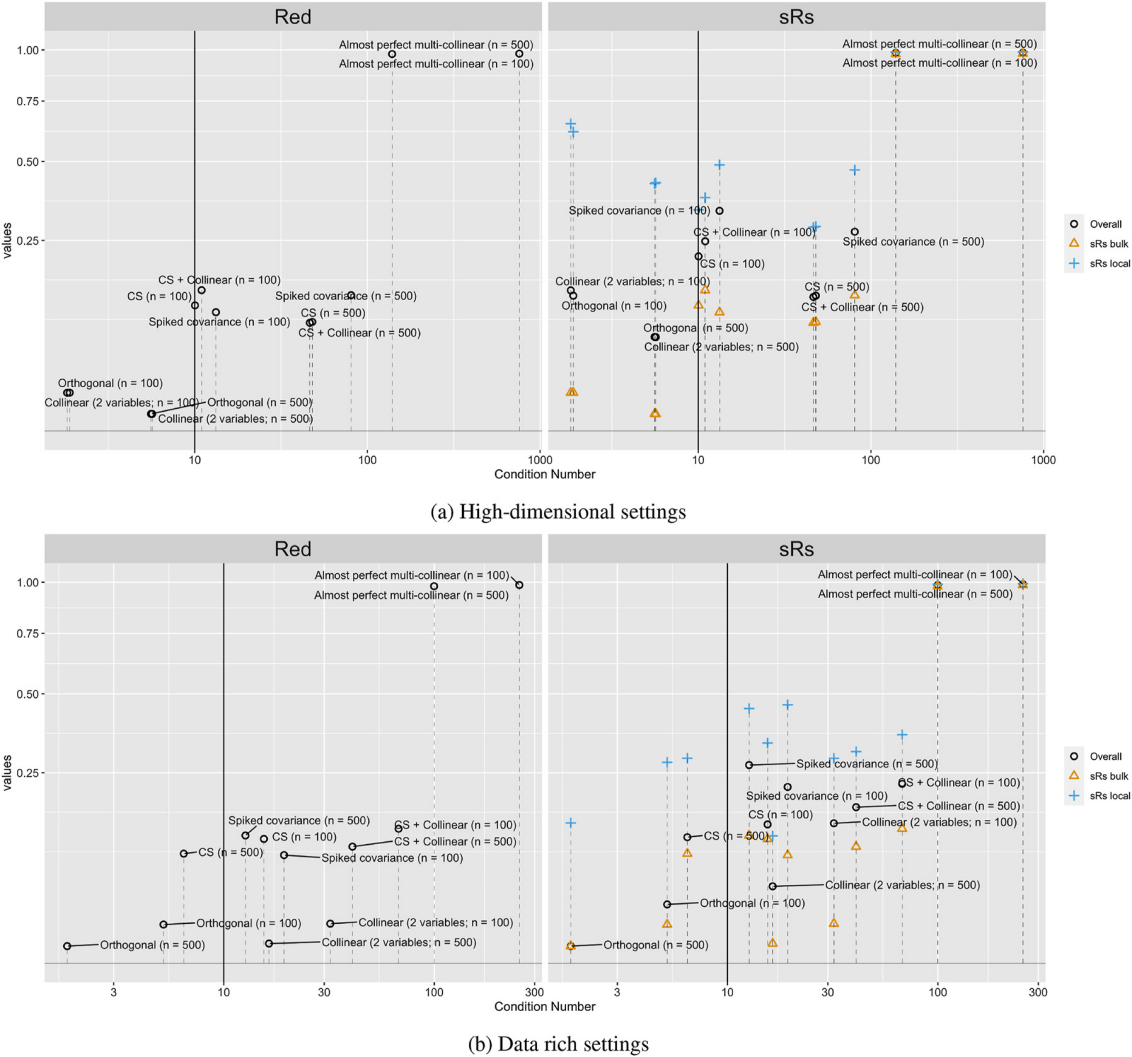
Measuring overall severity of multi-collinearity using condition number, *Red* indicator and *sRs*. The vertical line marks the condition number cut-off at 10 to suggest presence of possible multi-collinearity.

We compared the *Red* indicator and the proposed overall measure *sRs* Equation (2.8) to the condition number for assessing severity of multi-collinearity (Figure 2a). Unsurprisingly, the size of the condition number did not fully correspond to the severity of multi-collinearity under the impact of spurious correlations associated with high-dimensional data. In all scenarios, the *Red* indicator was identical to *BsRs*, but smaller than the overall measure *sRs* (Figure S1). But under *n* < *p*, when data matrices are necessarily under rank, and the *LsRs* component received a boost through the trailing singular values that were very close to zero. Indeed, the main advantage *sRs* had over the *Red* indicator and condition number is its sensitivity to near-collinearity *ρ* = 0.99 due to the added *LsRs* component. This contrast was expected since a global measure of “averaged linear relationship” might be less sensitive to local collinearity, for example, when two variables are near collinear. Given a fixed sample size, *Red* ranked the compound symmetry structure to be less severely multi-collinear than the spiked covariance structure as opposed to the other way around for *sRs*. This is because *sRs* puts more weight on the bulk of correlations through their contributions to *d*_1_, as well as locally strong correlation through their influences on *d*_*p*_ or *d*_*n*−1_. In the case of a low rank structure, the larger *sRs* was due to the strong regional (between local and bulk) correlations, which contributed to the first a few leading singular values. In contrast, multi-collinearity under a compound symmetry structure has only one leading singular value, and thus the advantage of *sRs* was less pronounced.

#### 3.2.2 Data rich settings

When *n* > *p*, *sRs* showed better agreement with the commonly used condition number than *Red* as reflected by points being closer to the line of reference, especially for the detection of two collinear variables (Figure 2b). On the other hand, *Red* was unable distinguish the scenarios of a compound symmetric covariance and that combined with two near collinear variables. Notice that a compound symmetric covariance with *ρ* = 0.3 is not considered to have a concerning level of multi-collinearity as these are *p* very weak collinear relationships, all of the same size.

**Figure 3:**
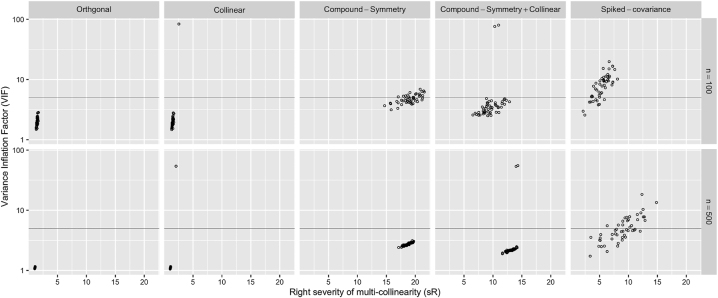
A scatterplot of *VIF*_*j*_ and *sR*_*j*_ under data rich settings. The horizontal line indicates a detection threshold of 5 for *VIF*_*j*_.

In terms of individualized measures, though *VIF*_*j*_ and *sR*_*j*_ were designed to capture slightly different features of data, they did correlate to some extent, especially when sample size is large (*n* = 500; Figure 3). As discussed in Supplementary Section 6, the numerical difference between the two measures is due to the joint correlation structure in the remaining *p* − 1 variables. The results in fact suggested these two measures are complementary to each other. Given the same *VIF*_*j*_ value by varying the remaining *p* − 1 variables, the pairwise correlation of *j*th variable with each of the *p* − 1 variables can vary. For example, two variables having the same *VIF*_*j*_ value means they can be equally explained by the other *p* − 1 variables. At the same time, the same two variables could have similar or very different *sR*_*j*_ values, with a larger *sR*_*j*_ suggesting the involvement of a larger number of individually weak relationships and a smaller *sR*_*j*_ suggesting the involvement of a few, but stronger relationships.

### 3.3 Computational considerations

Computation of the *sR*_*j*_ statistic can be formulated in several algebraically equivalent ways that vary in computational cost and interpretability. For each individual measure, Equation (2.2) shows that the sum of pairwise squared correlation coefficients is mathematically equivalent to a weighted sum of squared singular vector loadings, where the weights are proportional to the fourth powers of the singular values. Equation (6.2) further connects *sR*_*j*_ to the VIF regression framework, though this OLS formulation is by far the most computationally demanding.

The pairwise correlation implementation computes only the required *p*(*p* − 1)/2 correlations, yielding an exact result with *O*(*np* ^2^) time and *O*(*p* + *n*) memory. The SVD-based computation relies on a spectral decomposition of the data matrix $X\in R^{n\times p}$ , with computational complexity *O*(*np* ^2^) when *p* ≤ *n*, or *O*(*n* ^2^ *p*) when *p* > *n*. Memory usage is dominated by storing *X* and the full right singular vectors *V*, leading to *O*(*np* + *p* ^2^) memory complexity. While more algebraically compact than pairwise correlation, the full SVD becomes limiting in high-dimensional settings, motivating the use of truncated or randomized variants. A truncated randomized SVD approach (e.g., irlba, with *k* = 100) reduces the computational cost to *O*(*npk*), with a corresponding reduction in memory usage.

The OLS-based formulation is equivalent to computing VIF for each variable, requires *p* separate regressions of one variable on all others. This results in a total time complexity of *O*(*np* ^3^), and memory scaling with *O*(*np*). While exact and interpretable, this approach becomes computationally prohibitive for *p* > 1,000, and was excluded from benchmarks.

To understand the empirical computational cost, we focused on the pairwise correlation and SVD-based formulations, which represent the most direct and the most algebraically compact implementations, respectively. Benchmarking was conducted across the range of configurations reflecting small to very large high-dimensional settings. The configurations included combinations of sample size *n* ∈ {1,000, 2,000, 5,000} and variable count *p*:(*p*) ∈ {1,000, 5,000, 10,000, 50,000, 100,000}. For high-dimensional settings (*p* > = 10,000), where storing the full correlation matrix or computing a full SVD becomes infeasible, we implement approximate methods based on truncated randomized SVD (“irlba” at *k* = 100) and memory-efficient data structures via “bigstatsr” Filebacked Big Matrix objects.

All computations were performed on the Digital Alliance of Canada high-performance computing clusters. Each benchmarking run was submitted as a separate SLURM job with up to 256 GB of memory and a 20-h wall time limit. No GPUs were used.

For small to moderately sized applications, the pairwise correlation-based approach offers the most memory-efficient and exact computation of *sR*_*j*_, and is tractable across a wide range of dimensions when runtime is not a concern (Figure S2). However, for ultra high-dimensional settings (e.g., *p* = 100,000), this approach becomes impractical due to excessive runtime, and in our benchmarking, jobs exceeded the 20-h wall time and memory limit without completion. The truncated SVD formulation provides a highly scalable approximation that substantially reduces runtime requirements, while emphasizing global multicollinearity patterns driven by top singular directions. This makes it well-suited when sensitivity to local dependencies is less critical, or when working with ultra high-dimensional data.

## 4 Application to the 1000 genomes project data

The population genetics equivalent of multi-collinearity is linkage disequilibrium (LD), reflecting correlation between different genetic markers. For any pair of bi-allelic markers, the LD can be quantified by the squared Pearson’s correlation coefficient. LD can be interpreted at the genome-wide scale to reflect population history, breeding system and the geographic subdivision within human populations (Slatkin 2008). At the same time, it can be viewed at a regional level indicating influences from selection, mutation and gene conversion (Slatkin 2008). Thus, as the number of genetic markers involved increased, the large numbers of pairwise Pearson’s correlation coefficients make the studying of LD pattern over genomic regions of arbitrary size a challenging task.

The 1000 Genomes Project (1000 Genomes Project Consortium and others 2015) is a well-established reference for genetic variations and contains samples from several continental and sub-region populations. We applied the individual-valued uBVA and *sRs*, along with *LsRs* and *BsRs*, measures to understand the severity of multi-collinearity within genetically homogeneous populations, as well as contrasting these measures across populations. The univariate *sR*_*j*_ allows the comparison at each genetic marker, while the overall measures can be used to inform the overall burden of multi-collinearity.

### 4.1 Data information and quality controls

Standard quality controls on the genotype data are outlined in Roslin et al. (2016) and the data are publicly available (http://www/tcag.ca/tools/1000genomes.html). This set of data contains individuals from Africa (AFR; *n* = 353), East Asia (EAS; *n* = 480), Europe (EUR; *n* = 522), South Asia (SAS; *n* = 100), and the Latin America (AMR; *n* = 269). The analyses were restricted to bi-alleleic markers on autosomes. For each continental population, we applied additional data filtering steps to exclude single nucleotide polymorphisms (SNPs) with minor allele frequency (MAF) less than 0.01, with any missingness, and Hardy-Weinberg Equilibrium *p*-value <1E−5 . To harmonize the analysis in the combined sample, we retained only SNPs present in all continental populations, leaving 193,744 SNPs in the analysis, representing 20–50 % of the SNPs originally available in each population. The genomic coordinates are based on the GRCh37/hg19 build. As a control step, we produced the first two genetic principal components using the subset of overlapping SNPs, and confirmed that they are sufficient to stratify samples at the continental and sub-population level (Figure S4).

### 4.2 Chromosome specific patterns of multi-collinearity

**Figure 4:**
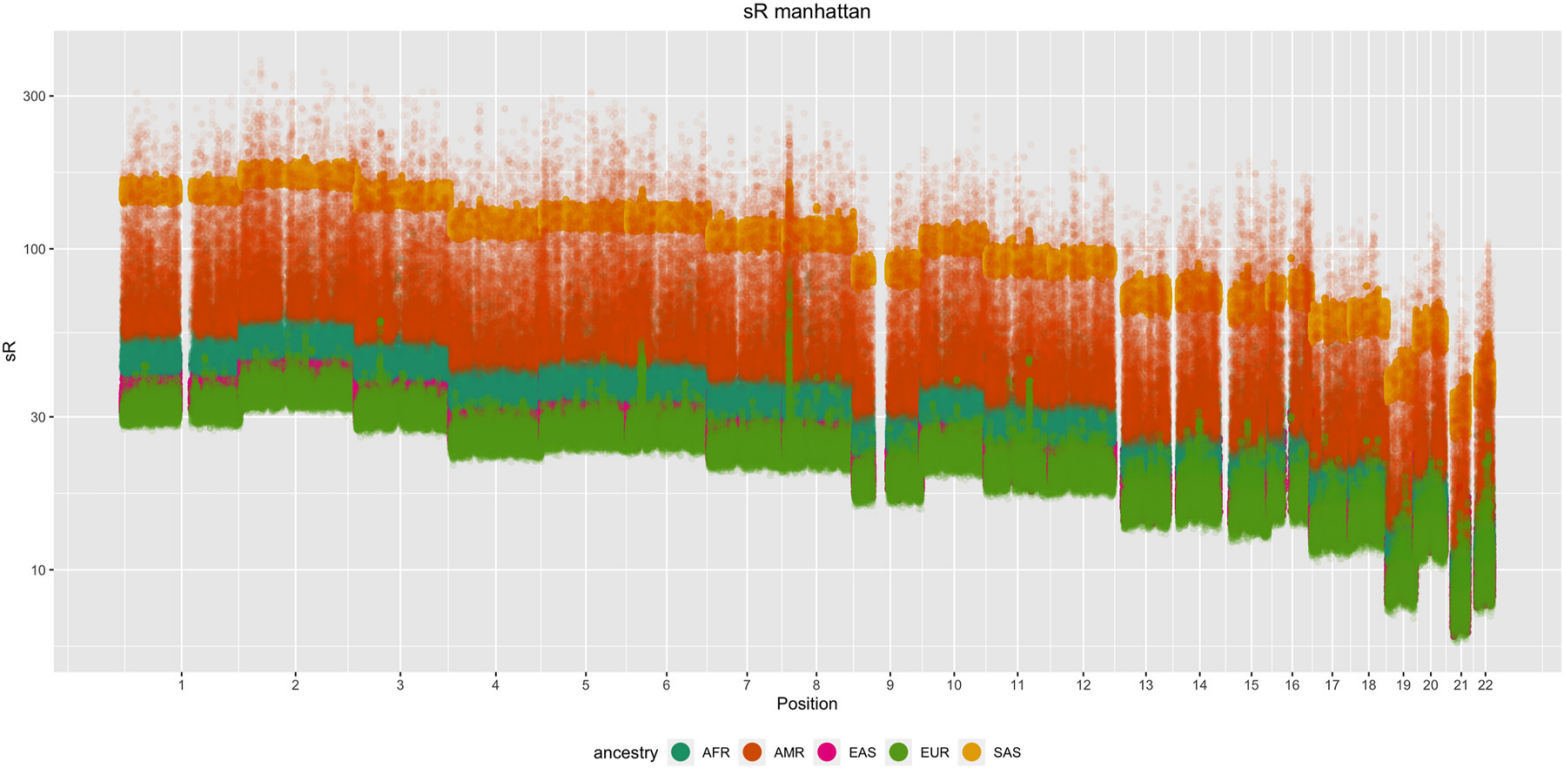
A manhattan type plot for {*sR*_*j*_} as a function of the genomic location within each chromosome.

The uBVA measures (i.e. {*sR*_*j*_}) were calculated for each chromosome separately and presented in a similar manner to a Manhattan plot (Figure 4), typical for genome-wide applications. We expected them to roughly follow the chromosome size (number of SNPs), but with varying peaks and valleys highlighting specific regions of high/low local multi-collinearity. Interestingly, there are a large number of visible peaks in the Europeans, only some of them are shared with other populations, in particular those well-known long range LD regions at chromosome 6, 8 and 11 (Price et al. 2008) with slight differences in the exact location for different populations. For example, the region identified on chromosome 6 overlaps with the human leukocyte antigen (HLA) region. In Europeans, the peak region ranged from 25.9 to 32.7 MB, similar to the 26.4–33.5 MB in East Asians, but was much shorter in South Asians, only between 31.0 and 32.3 MB.

In general, East Asian, European and South Asian have sporadic long-range LD regions, represented by the occasional peaks in Figure 4, while populations in Latin America and Africa seemed to have more complex patterns of long and short range LD, corroborating the findings in Park (2019).

We observed two types of collective patterns of {*sR*_*j*_} across chromosomes and populations: those from populations in East Asia, Europe, and South Asian can be classified as being roughly symmetric (Figures S12, S6, and S8) and those from Africa and Latin America tended to have heavier tails for most chromosomes (Figures S4, S10). The shift in the overall distribution should not be heavily influenced by outliers, such as the presence of a few long range LDs regions or strong LD blocks. Rather, considering the high-level of admixture in these populations, we hypothesized that these were probably the result of enriched genetic diversity manifested as a handful of large eigenvalues within each population (Figures S5, S13, S7, S11, and S9).

### 4.3 Genome-wide summaries of multi-collinearity

We then examined the overall level of multi-collinearity using genome-wide data (all 193,744 SNPs) and the results suggested the majority of multi-collinearity patterns were due to local relationships rather than global. The *RED* indicator gave a slightly higher level of “averaged correlation” in South Asia population and a lower level in Europeans and East Asians (Table 1). On the other hand, *sRs* offered better granularity in the type of forces driving the averaged correlation. Specifically, though both Europeans and East Asians had similar *RED* values, their *sRs* and *LsRs* values collectively suggested a stronger local multi-collinearity in Europeans than in East Asians (Table 1). This pattern aligns with known differences in LD structure across populations, where European populations tend to exhibit higher local correlation due to longer haplotype blocks and lower effective recombination rates in certain genomic regions (Berisa and Pickrell 2015). Differences in local versus global patterns of genetic multicollinearity have important implications for polygenic score transferability. Genetic signals identified in European populations often fail to replicate in non-European populations, especially in population of African ancestry, where lower LD reduces tagging of causal variants, leading to diminished polygenic predictive performance. Similarly, fine-mapping in European populations may yield inflated credible sets that do not resolve causal variants due to strong local multi-collinearity. These findings reinforce the need for ancestry-aware models of multicollinearity and LD structure, and including diverse populations in genetic studies to improve the resolution, reproducibility, and equity of genomic inference.

**Table 1:** A genome-wide summary of multi-collinearity for each continental population and the combined samples.

	RED	sRs	LsRs	BsRs
Combined	0.01325	0.00180	0.00451	0.00018
SAS	0.02365	0.01298	0.03674	0.00056
EAS	0.01140	0.00396	0.01187	0.00013
AFR	0.01323	0.00485	0.01465	0.00017
AMR	0.01792	0.00363	0.00826	0.00032
EUR	0.01092	0.00506	0.01608	0.00012

## 5 Discussion

Originally intended for detecting multi-collinearity under *n* > *p*, the *Red* indicator is equally adaptable to rank the severity of multi-collinearity in high-dimensional settings (*n* < *p*). As a measure of “averaged” correlation in the data, *Red* is sensitive to multi-collinearity that severely affects a large number of variables, but tends to ignore strong local relationships in the presence of moderate bulk relationships. Indeed, unlike *sRs*, *Red* does not have a mechanism to distinguish local and bulk relationships. On the other hand, the condition number is perhaps more specific to detect ill-posed problems as it is directly related to the numerical accuracy of the inverse of *X* ^*T*^ *X*. Though these measures are sometimes useful as indicators of the overall multi-collinearity, they fall short in generating variable-specific information. As compared to *sR*_*j*_, the individual-valued *VIF*_*j*_ can also identify specific variables involved in ill-conditioned problems and is capable of harvesting both local and global linear relationships, but can only be applied when *n* < *p*. In conclusion, the proposed *sRs*, *BsRs* and *LsRs*, combined with {*sR*_*j*_} are recommended as measures of multi-collinearity in high-dimensional settings. To support interpretation in high-dimensional settings, we propose a practical threshold based on simulation results in Section 3. Specifically, we recommend a cutoff of 0.3 for the overall *sRs*, as well as for its bulk (*BsRs*) and local (*LsRs*) components, based on their unweighted formulations.

We want to highlight some potential improvements that are of interest for future research work. Firstly, *sR*_*j*_ are empirical measures and a natural next step is to leverage theoretical results from random matrix theory to further derive their statistical properties. Secondly, it would be of interest to construct two-sample or multiple-sample statistical tests for quantities such as *sRs*, *BsRs* and *LsRs*, thus enabling a formal statistical comparison of the severity and sources of multi-collinearity. Finally, though the application to autosomal markers yielded insightful results, the same might not translate to the X-chromosome due to differences in the number of chromosomal copies between sexes. One of the complications is that since females carry two copies and males carry only one copy of the X-chromosome, the multi-collinearity measures derived from the observed data are expected to vary with respect to the sex ratios. As a result, though the measures are still valid in the sense that they can be computed and reflect the observed severity of multi-collinearity, they cannot be reliably used to compare LD patterns between samples.

It is worth noting that similar measures to *sR*_*j*_ have been proposed in genetic applications: *LDadj* was used in the construction of polygenic risk scores (PRS) for prediction (Pare et al. 2016, 2017), and *LDscore* was used to demonstrate the polygenicity of a trait, such as in LD-score regression (Bulik-Sullivan et al. 2015). Both are in fact truncated versions of the *sR*_*j*_ and are denoted by the sum of squared Pearson’s correlation coefficients:

(5.1) $${LDadj}_{\left(j\right)}=\overset{j+t}{\underset{j^{\prime }=j-t}{\sum }}r_{j^{\prime }j}^{2},$$

and

(5.2) $${LDscore}_{\left(j\right)}=\overset{j+t}{\underset{j^{\prime }=j-t}{\sum }}r_{j^{\prime }j}^{2}-\frac{1-r_{j^{\prime }j}^{2}}{n-2},$$

where *LDscore* has an additional term such that each squared Pearson’s correlation remains unbiased (under *n* > *t*). The value of *t* is defined by assuming the neighbouring *t* genetic variants up- and down-stream are sufficient to capture the local LD (or covariance) structure, thus the choice is subjective. A window of radius 1 centiMorgan around the index variant was recommended (Bulik-Sullivan et al. 2015). Contrary to uBVA, *LDadj* or *LDscore* do not intend to capture the systematic effect, but only the local effect of multi-collinearity. Consequently, these truncated measures are restricted to analyses within a homogeneous population and are not tailored for comparisons across samples of distinct populations; further, since the truncation occurs through a rolling window, the measures are not directly comparable from variable to variable.

## 6 Concluding remarks

Our multi-collinearity measures {*sR*_*j*_} offer an alternative univariate perspective to visualize multi-collinearity patterns. They also enable the construction of a high-level summary measure *sRs* that sheds light on the sources of multi-collinearity through the relative contribution from *LsRs* and *BsRs*, which can inform the choice of an appropriate data learning strategy. The fact that these can be applied regardless of data dimensions is an attractive feature in high-dimensional data applications. Besides providing a visual inspection and numerical summary of multi-collinearity in high-dimensions, the proposed measures are amendable to various downstream analyses and potential applications, for example, as informative shrinkage weights to construct high-dimensional estimators. Finally, the simplicity in their construction also enables convenient data sharing for open science research.

## Supplementary Material

Supplementary Material Details
