# Supplementary material for: Perfect collinearity not created equal: measuring and visualizing the severity of multi-collinearity of modern omics data
Source: Stat Appl Genet Mol Biol. 2026 Feb 10;24(1):20250043. doi: 10.1515/sagmb-2025-0043 (PMC12909097; doi:10.1515/sagmb-2025-0043)
Supplement: Supplementary file 1 — Supplementary Material Details [file j_sagmb-2025-0043_suppl_001.pdf]

SUPPLEMENTARY MATERIAL FOR "PERFECT COLLINEARITY NOT CREATED EQUAL: MEASURING AND VISUALIZING THE SEVERITY OF MULTI-COLLINEARITY OF MODERN OMICS DATA"

TECHNICAL REPORT

Wei Q. Deng
Department of Psychiatry and Behavioural Neurosciences
McMaster University
Peter Boris Centre for Addictions Research
St. Joseph's Healthcare Hamilton
Hamilton, Canada
dengwq@mcmaster.ca

Radu V. Craiu
Department of Statistical Sciences
University of Toronto
Toronto, Canada
radu.craiu@utoronto.ca

Lei Sun
Department of Statistical Sciences
Dalla Lana School of Public Health
University of Toronto
Toronto, Canada
sun@utstat.toronto.edu

1 Proofs

Proof of property 2.1

Proof. : Since XX^TXX^T is a square matrix with non-negative eigenvalues $d_1^4, \ldots, d_{n-1}^4$, the trace is simply the sum of eigenvalues:

$$\text{tr}(XX^TXX^T) = \sum_{i'=1}^{n-1} d_{i'}^4.$$

It then follows from the cyclic property of trace that:

$$\sum_{j=1}^{p} SR_j = \text{tr}(X^TXX^TX) = \text{tr}(XX^TXX^T) = \text{tr}(UD^4U^T) = \sum_{i=1}^{n} SL_i.$$

Proof of property 2.2

Proof. :

$$\text{diag}[\text{cor}(X)^2]_j = \text{diag}\left[\frac{X^TXX^TX}{(n-1)^2}\right]_j = \frac{SR_j}{(n-1)^2},$$

and

$$\text{diag}[\text{cor}(X)^2]_j = \sum_{j'=1}^{p} r_{jj'}^2.$$

Proof of property 2.3

Proof. : Following the Cauchy-Schwarz inequality, the lower bound is given by:

$$\begin{aligned} SR_j &= \sum_{i'=1}^{n-1} v_{ji'}^2 d_{i'}^4 = \sum_{i'=1}^{n-1} \frac{(v_{ji'}^2 d_{i'}^2)^2}{v_{ji'}^2} \\ &\geq \frac{(\sum_{i'=1}^{n-1} v_{ji'}^2 d_{i'}^2)^2}{\sum_{i'=1}^{n-1} v_{ji'}^2} \\ &= \frac{(n-1)^2}{\sum_{i'=1}^{n-1} v_{ji'}^2} \geq (n-1)^2, \end{aligned}$$

while the upper bound is:

$$\begin{aligned} SR_j &= \sum_{i'=1}^{n-1} v_{ji'}^2 d_{i'}^4 \\ &\leq d_1^2 \sum_{i'=1}^{n-1} v_{ji'}^2 d_{i'}^2 \\ &= d_1^2(n-1). \end{aligned}$$

Proof of Lemma 2.1

Proof. Since SR_j is simply the jth diagonal element of X^TXX^TX, we approach this by calculating the expected value of $SR_j = e_j^TX^TXX^TXe_j$, where $e_j = (0, \ldots, 0, 1, 0, \ldots, 0) \in \mathbb{R}^p$ is a standard basis vector with value 1 at the jth place. Further, it follows that $(n-1)\hat{\Sigma} = X^TX \sim \mathcal{W}_p(\Sigma, n)$ has a Wishart distribution with parameters Σ and degrees of freedom n.

Following Proposition S1 of [Dicker, 2014], where explicit expressions for the expectation of moments of a Wishart random matrix were derived, it is easy to write down the expectation of SR_j as:

$$\begin{aligned}\mathrm{E}(e_j^T X^T X X^T X e_j) &= p(n-1)\frac{\mathrm{tr}(\Sigma_p)}{p} e_j^T \Sigma_p e_j + n(n-1) e_j^T \Sigma_p \Sigma_p e_j \\ &= (n-1)(\Sigma_p)_{jj}\mathrm{tr}(\Sigma_p) + n(n-1)(\Sigma_p)_{.j}^T(\Sigma_p)_{.j}.\end{aligned}$$

The expectation can be further simplified if the true covariance Σ has diagonal elements 1:

$$\mathrm{E}(SR_j) = (n-1)p + n(n-1)\Sigma_j^T \Sigma_j.$$

□

2 Relationship with existing measures of multi-collinearity

Since measures are often derived from sample eigenvalues, which are closely related to singular values, here we reveal the relationship between proposed and existing measures of multi-collinearity.

The *Red* indicator Both the *Red* indicator [Kovács et al., 2005] and sR_j can be used without dimension restrictions and it turns out the two are closely related:

$$\mathrm{Red} = \sqrt{\frac{\mathrm{tr}[X^T X X^T X - (n-1)^2 I_p]}{p(p-1)(n-1)^2}} = \sqrt{\frac{\sum_{j=1}^p sR_j - p}{p(p-1)}},$$

where *Red* $\in [0,1]$ and $sR_j \in [1,p]$. It is regarded as a global measure of average correlation in the data over all pairwise variables or the proportion of redundant information, with values closer to 1 indicating a large number of near or perfect multi-collinearity relationships and values closer to 0 indicating little evidence of multi-collinearity. Given how *Red* is defined, the authors did not provide a recommended threshold at which a concerning level of multi-collinearity is present.

In relation to *Red*, sR_j can be seen as its individual-level counterpart and is potentially more useful for contrasting variables for their relative involvement in multi-collinearity. It should emphasized that individual sR_j values alone cannot distinguish between the "bulk weak" and "local strong" scenarios as the same sR_j value could be given by many weak relationships or a few strong relationships. In reality, the ambiguity also remains for *Red* indicator values that are closer to the middle. For example, a *Red* value of 0.4 can be achieved by either a large number of weak collinear relationships or a small number of perfect or near collinear relationships, with the latter having a bigger impact on matrix solutions to linear regression problems.

Variance Inflation Factor (*VIF*) Though the *VIF* is restricted to the setting of $n > p$, it is still of interest to compare sR_j and *VIF*$_j$ on an equal footing as individual-valued measures. Note that when $n > p$, components of the SVD of X have different dimensions: $U \in \mathbb{R}^{n\times p}$, $V \in \mathbb{R}^{p\times p}$, and $D = \mathrm{diag}[d_1, \dots, d_p] \in \mathbb{R}^{p\times p}$. In this case, d_p does not equal to 0 following the column-wise mean and variance standardization. It should be noted that *VIF*$_j$ is only suitable when the data matrix is full rank, while sR_j can be calculated without such restriction.

The *VIF* of the jth predictor can be expressed as:

$$\begin{aligned}\mathit{VIF}_j &= \frac{1}{1-R_j^2} \\ &= \frac{x_j^T x_j}{x_j^T x_j - x_j^T X_{-j}(X_{-j}^T X_{-j})^{-1} X_{-j}^T x_j} \\ &= \frac{1}{1-(n-1)b_{-j}^T (X_{-j}^T X_{-j})^{-1} b_{-j}},\end{aligned} \tag{2.1}$$

where x_j denotes the jth column of X, X_{-j} the data matrix with jth column removed, and $b_{-j} = \frac{1}{n-1}X_{-j}^T x_j$ the vectorized univariate regression coefficients estimated between the jth variable and each of the other $p-1$ variables. Using the same notation, the multivariate regression coefficients estimated using the jth variable as the response and the other $p-1$ variables as the predictors can be expressed as $(n-1)b_{-j}^T(X_{-j}^T X_{-j})^{-1}b_{-j}$.

The proposed measure sR_j can be similarly expressed:

$$\begin{aligned} sR_j &= \sum_{j'=1}^{p} r_{jj'}^2 \\ &= \Big[1 + \sum_{j' \neq j} (\frac{1}{n-1} x_{j'}^T x_j)^2\Big] \\ &= (1 + b_{-j}^T b_{-j}). \end{aligned} \tag{2.2}$$

Both VIF_j and sR_j are driven by b_{-j}, with the main difference being how $b_{-j}^T b_{-j}$ is weighted. Note that as $(X_{-j}^T X_{-j})^{-1}$ is capable of simultaneously modelling relationship among the other $p-1$ variables, VIF_j is expected to be more sensitive than sR_j at recognizing multi-collinearity that involves a large number of variables as each element of $b_{-j}^T b_{-j}$ merely describes the strength of a bivariate relationship. From the alternative expression of sR_j according to the definition via the singular values, we obtain

$$sR_j = (n-1)^{-2} \sum_{i'=1}^{p} v_{ji'}^2 d_{i'}^4 = d_1^2 (n-1)^{-2} \left[\sum_{i'=1}^{p} (v_{ji'}^2 d_{i'}^2) \frac{d_{i'}^2}{d_1^2} \right]. \tag{2.3}$$

Though each $d_{i'}$ is weighted towards sR_j, the collective behaviour of sR_j will be influenced by a large d_1 and therefore captures information in the condition indices $\{\frac{d_1}{d_{i'}}\}_{i'=1,\ldots,p}$.

An important aspect is the detection of variables involved in multi-collinearity, which often requires a hard detection threshold. For the individual sR_j, suppose the data matrix is column standardized, one possible threshold for sR_j could be due to property 2.2 combined with an approximated distribution for the sample Pearson's correlation coefficient given in Stuart et al. [1994]:

$$r = \frac{t}{\sqrt{n-2+t^2}},$$

where t is a random variable following Student's t-distribution with degrees of freedom $n-2$. This results holds approximately for large enough n and the pairs of variables are assumed to be uncorrelated. It can be shown that r^2 then follows a beta distribution with shape parameters $1/2$ and $(n-2)/2$, and $\mathrm{E}(r^2) = (n-1)^{-1}$. Thus, a possible threshold for departure from orthogonal columns using sR_j could be $\frac{p-1}{n-1} + 1$ by summing up the $p-1$ expected values of squared sample Pearson's correlation coefficients assuming the true pairwise correlation is zero throughout.

3 Connection to the effective sample size and effective number of variables

Typically, in a regression, correlated samples do not change mean estimation, but rather influence inference through increased variance. As a result, the same estimator under correlated samples should have a variance adjusted for the effective sample size. For correlated variables, an analogous concept is the effective number of variables, which serves as an upper bound for the effective degrees of freedom of a model (usually defined as the trace of the hat matrix connecting the response to its fitted values, e.g. $H = X(X^T X)^{-1} X^T$, for OLS regression).

Here we focus on the effective number of variables and the effective sample size, without referencing a model fitting procedure, and show that the proposed measure of multi-collinearity can be used to inform the maximum possible values for both. As a result of the dual (i.e. column and row) perspectives on a data matrix X, the same technique can be applied to either X or X^T provided that the respective columns or rows are standardized to have mean 0 and variance 1.

Given a row standardized X and $n > p$, the effective sample size as determined by the left severity measure is at most:

$$\sum_{i=1}^{n} \frac{1}{sL_i} \leq \sum_{i=1}^{n} \sum_{i'=1}^{p} u_{ii'}^2 = p.$$

Analogously, given a column standardized X and $n < p$, $\sum_{j=1}^{p} \frac{1}{sR_j}$ can be considered the effective number of variables. Further, it can be shown that $\sum_{j=1}^{p} \frac{1}{sR_j}$ is at most $n-1$ following property 2.3. To see this, for each j:

$$\frac{1}{sR_j} \leq \sum_{i'=1}^{n-1} v_{ji'}^2,$$

which suggests that:

$$\sum_{j=1}^{p}\frac{1}{sR_j}\leq\sum_{j=1}^{p}\sum_{i'=1}^{n-1}v_{ji'}^2=n-1.$$

Since $\frac{1}{sR_j}$ is a constant between $1/p$ and 1, it can be viewed as the amount of non-redundant information in a variable prior to model selection. Consider the extreme case when all variables were truly uncorrelated, but under the impact of spurious correlation in high dimensions, the maximum degrees of freedom becomes $\min(n,p)-1=n-1$, meaning every variable is equally important prior to model selection. The opposite scenario is when all variables completely correlate with each other, the maximum degrees of freedom reduce to 1. Though each variable is equally important, their relative importance would be scaled by $\frac{1}{p}$, meaning the model can include any one of the variables.

However, these two concepts are really two sides of the same coin arising from the execution of a row or column standardization. For convenience, briefly consider a doubly centred ($\sum_{i=1}^{n}x_{ij}=\sum_{j=1}^{p}x_{ij}=0$) and doubly standardized ($\sum_{i=1}^{n}x_{ij}^2=n-1$ and $\sum_{j=1}^{p}x_{ij}^2=p-1$) data matrix X.

Suppose $n>p$, assume a multivariate normal model for each row of X with independent samples:

$$x_i\overset{\text{iid}}{\sim}\mathcal{N}(0,\Sigma),\quad i=1,2,\ldots,n,$$

where $\Sigma\in\mathbb{R}^{p\times p}$. With the means removed, it follows that $\hat{\Sigma}=\frac{1}{n}X^TX$ has a scaled Wishart distribution with mean and variance

$$\text{E}(\hat{\Sigma})=\Sigma\quad\text{and}\quad\text{Var}(\hat{\Sigma})=\frac{1}{n}\Sigma^{(2)},$$

where $\Sigma_{jk,lh}^{(2)}=\Sigma_{jl}^{(2)}\Sigma_{kh}^{(2)}+\Sigma_{jh}^{(2)}\Sigma_{kl}^{(2)}$ and $\Sigma^{(2)}\in\mathbb{R}^{p^2\times p^2}$.

Similarly, suppose $n<p$, a multivariate normal model can be assumed for each column of X with independent variables:

$$x_j\overset{\text{iid}}{\sim}\mathcal{N}(0,\Phi),\quad j=1,2,\ldots,p,$$

where $\Phi\in\mathbb{R}^{n\times n}$. It follows that $\hat{\Phi}=\frac{1}{p}XX^T$ has a scaled Wishart distribution with mean and variance

$$\text{E}(\hat{\Phi})=\Sigma\quad\text{and}\quad\text{Var}(\hat{\Phi})=\frac{1}{p}\Phi^{(2)},$$

where $\Phi_{jk,lh}^{(2)}=\Phi_{jl}^{(2)}\Phi_{kh}^{(2)}+\Phi_{jh}^{(2)}\Phi_{kl}^{(2)}$ and $\Phi^{(2)}\in\mathbb{R}^{n^2\times n^2}$.

Following Theorem 8.4 of Efron [2012], when rows of X are not independent and $n>p$, the effective sample size n_{eff} is defined by

$$n_{\text{eff}}=\frac{n}{1+(n-1)\left[\frac{n\sum_{i'=1}^{p}d_{i'}^4}{n(n-1)p^2}-\frac{1}{n-1}\right]}=\frac{n^2p^2}{\sum_{i'=1}^{p}d_{i'}^4}=\frac{n^2}{\sum_{i=1}^{n}sL_i}. \tag{3.1}$$

In comparison, when columns of X are not independent and $n<p$, the effective number of variables (p_{eff}) is defined by

$$\text{p}_{\text{eff}}=\frac{p}{1+(p-1)\left[\frac{p\sum_{i'=1}^{n-1}d_{i'}^4}{n^2p(p-1)}-\frac{1}{p-1}\right]}=\frac{n^2p^2}{\sum_{i'=1}^{n-1}d_{i'}^4}=\frac{p^2}{\sum_{j=1}^{p}sR_j}. \tag{3.2}$$

The inequality of arithmetic and geometric means implies

$$\frac{p^2}{\sum_{j=1}^{p}sR_j}\leq\sum_{j=1}^{p}\frac{1}{sR_j},$$

and

$$\frac{n^2}{\sum_{i=1}^{n}sL_i}\leq\sum_{i=1}^{n}\frac{1}{sL_i},$$

which shows that $\sum_{j=1}^{p}\frac{1}{sR_j}$ and $\sum_{i=1}^{n}\frac{1}{sL_i}$ are indeed the maximum possible values for the effective number of variables and effective sample size, respectively.

4 Supplementary Figures

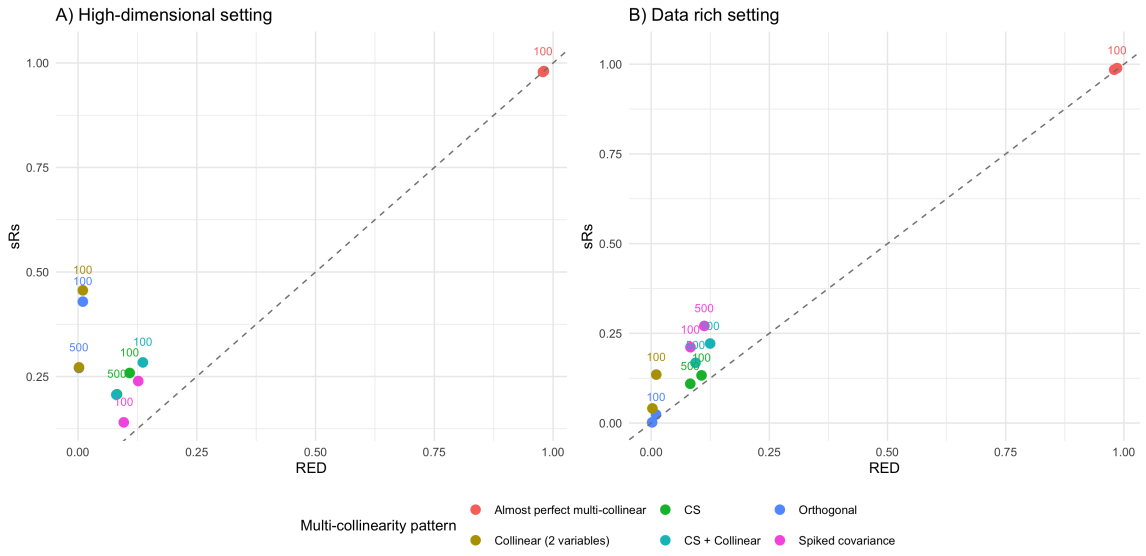

Figure S1: Scatterplot of RED vs. sRs highlights scenarios of divergent sensitivity to multi-collinearity patterns

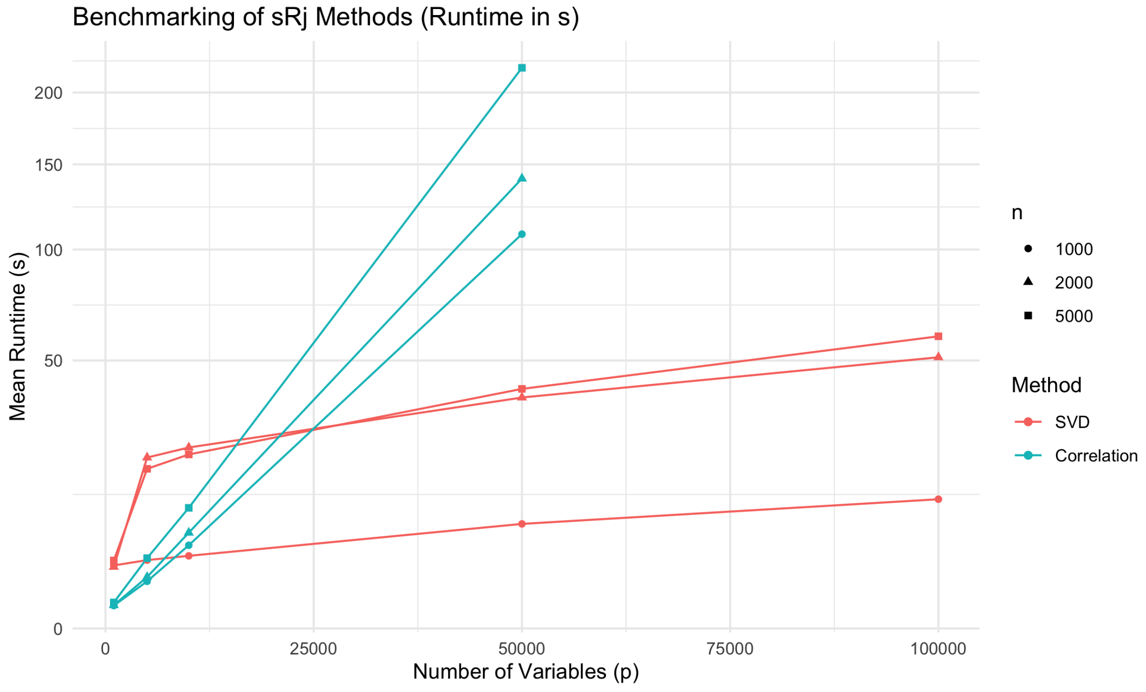

Figure S2: Benchmarking Computational Time for Multicollinearity Measures Across Matrix Dimensions

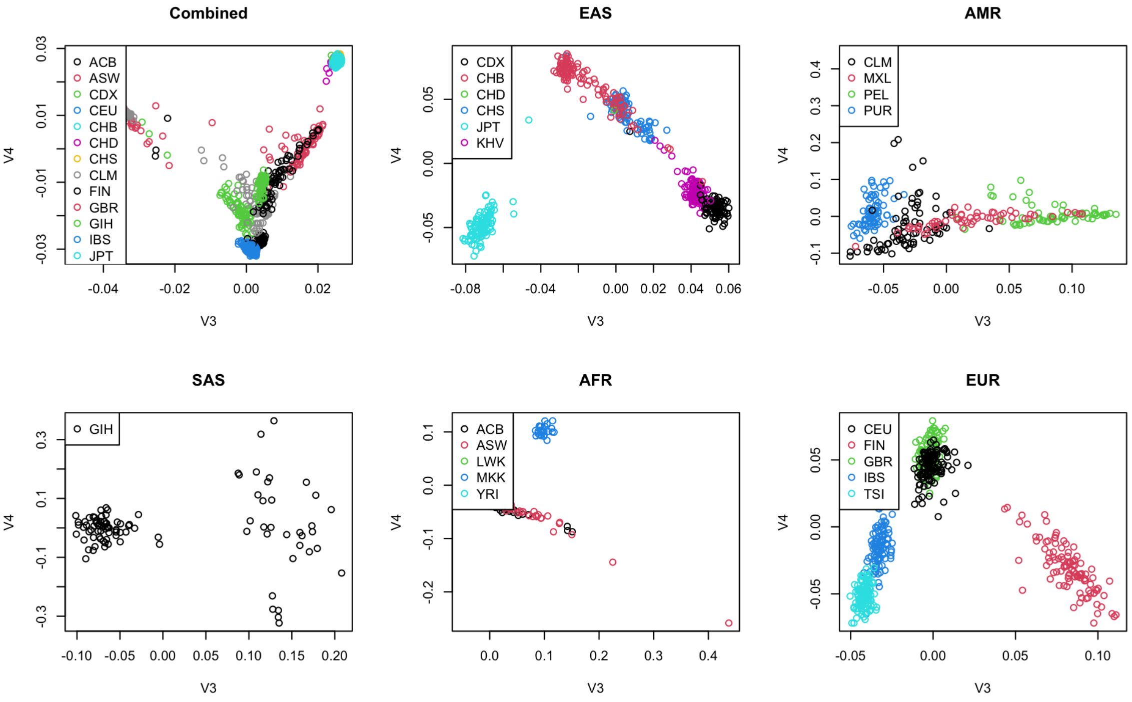

Figure S3: Scatterplots of the first genetic principal components for the combined and each continental populations.

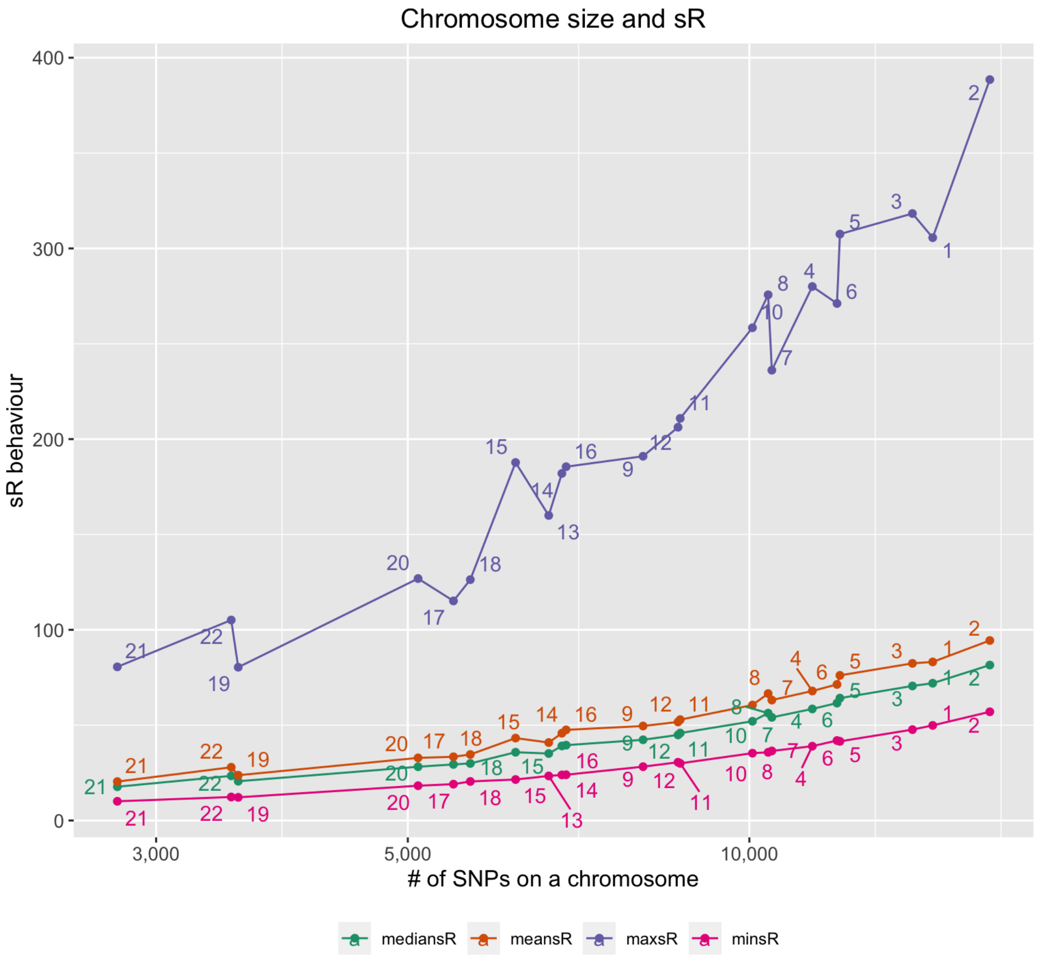

Figure S4: A summary of multi-collinearity as a function of chromosome size for populations in America.

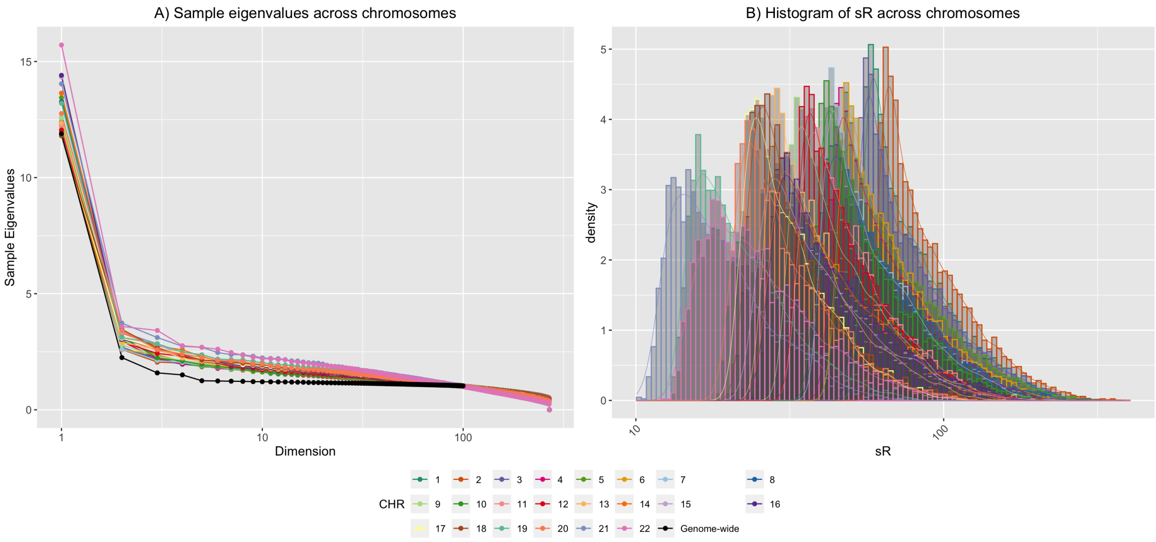

Figure S5: Patterns of multi-collinearity measured by $\{sR_j\}_j$ in America.

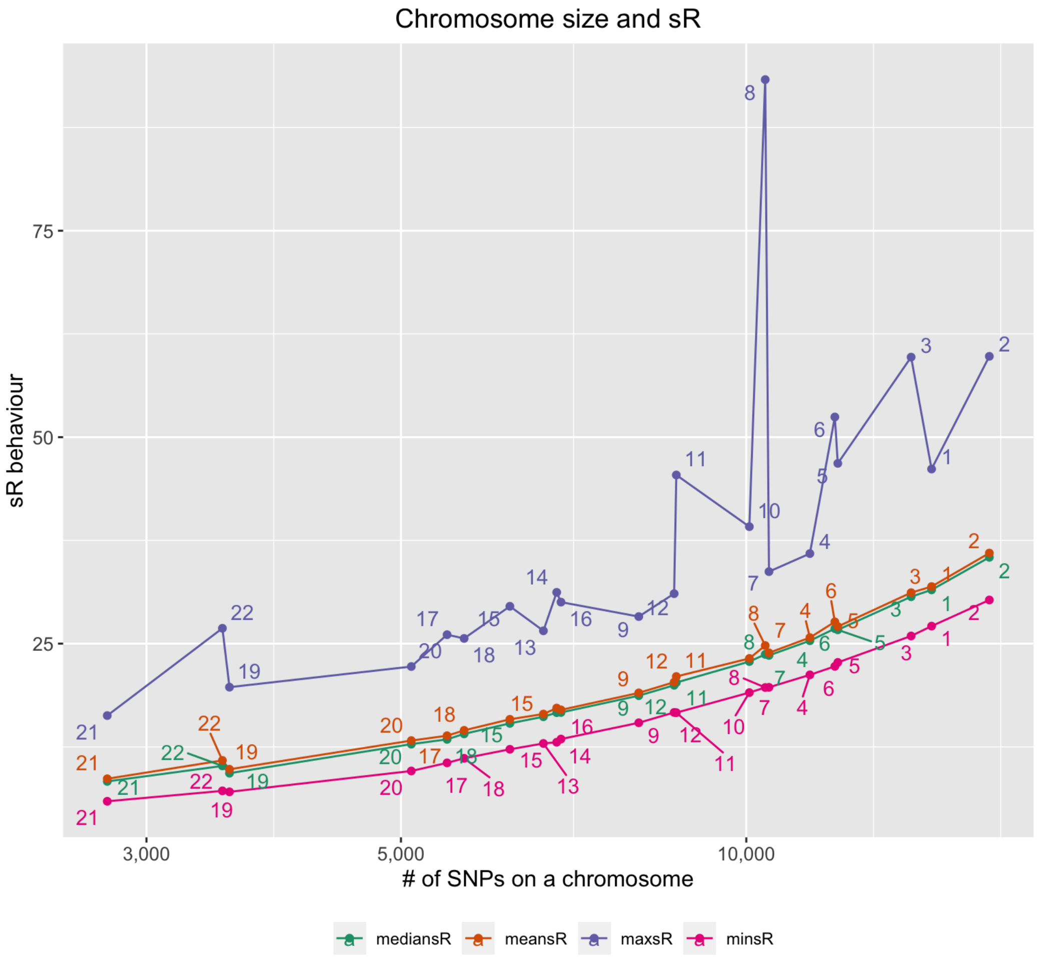

Figure S6: A summary of multi-collinearity as a function of chromosome size for populations in Europe.

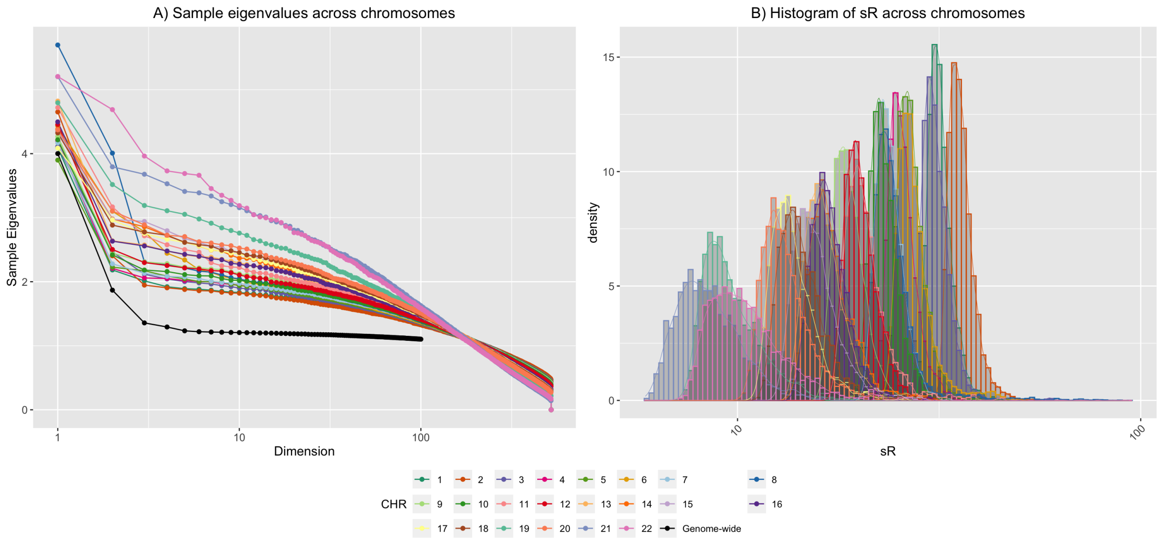

Figure S7: Patterns of multi-collinearity measured by $\{sR_j\}_j$ in Europe.

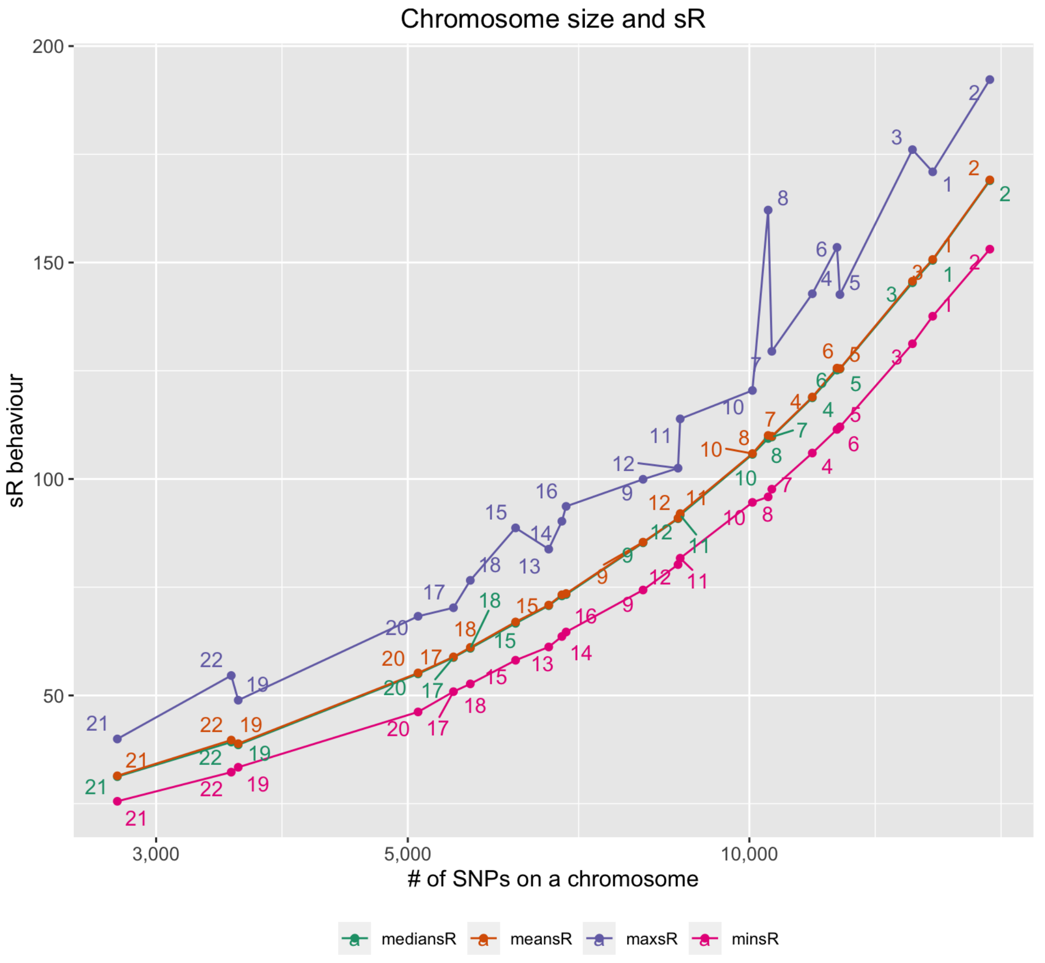

Figure S8: A summary of multi-collinearity as a function of chromosome size for populations in South Asia.

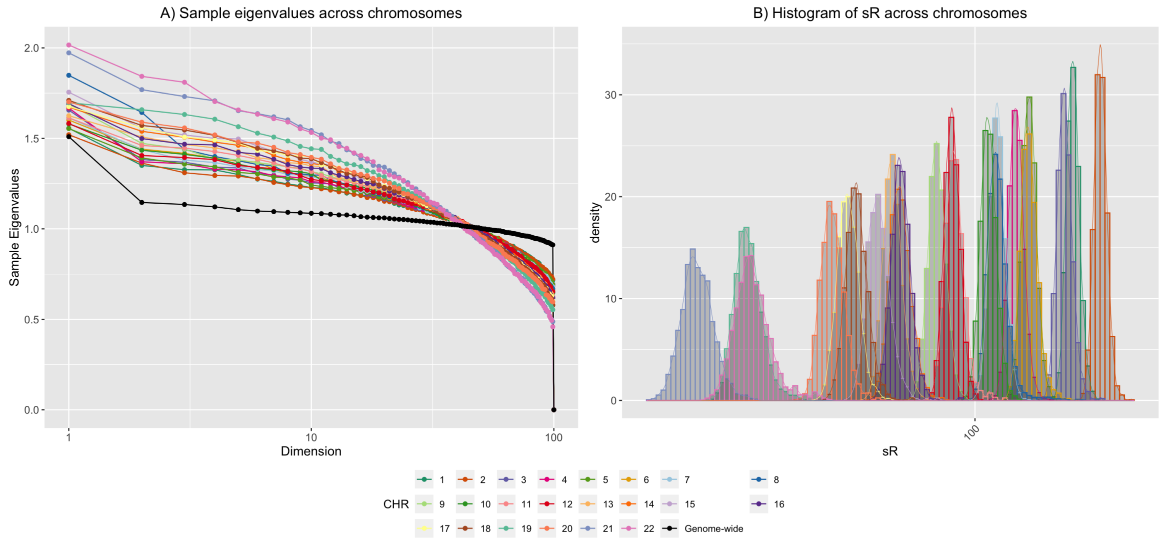

Figure S9: Patterns of multi-collinearity measured by $\{sR_j\}_j$ in South Asia.

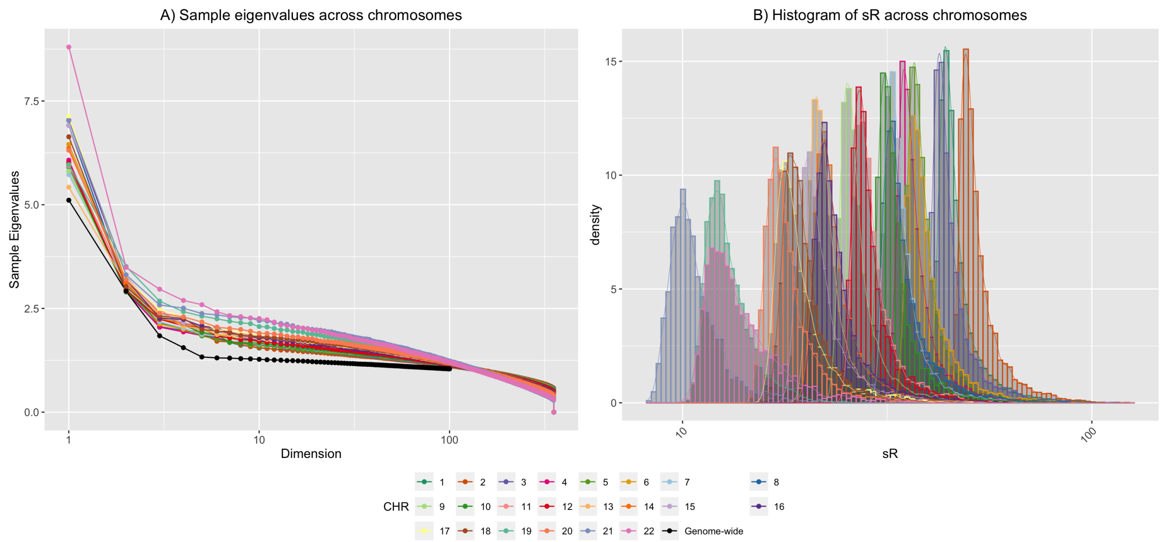

Figure S11: Patterns of multi-collinearity measured by $\{sR_j\}_j$ in Africa.

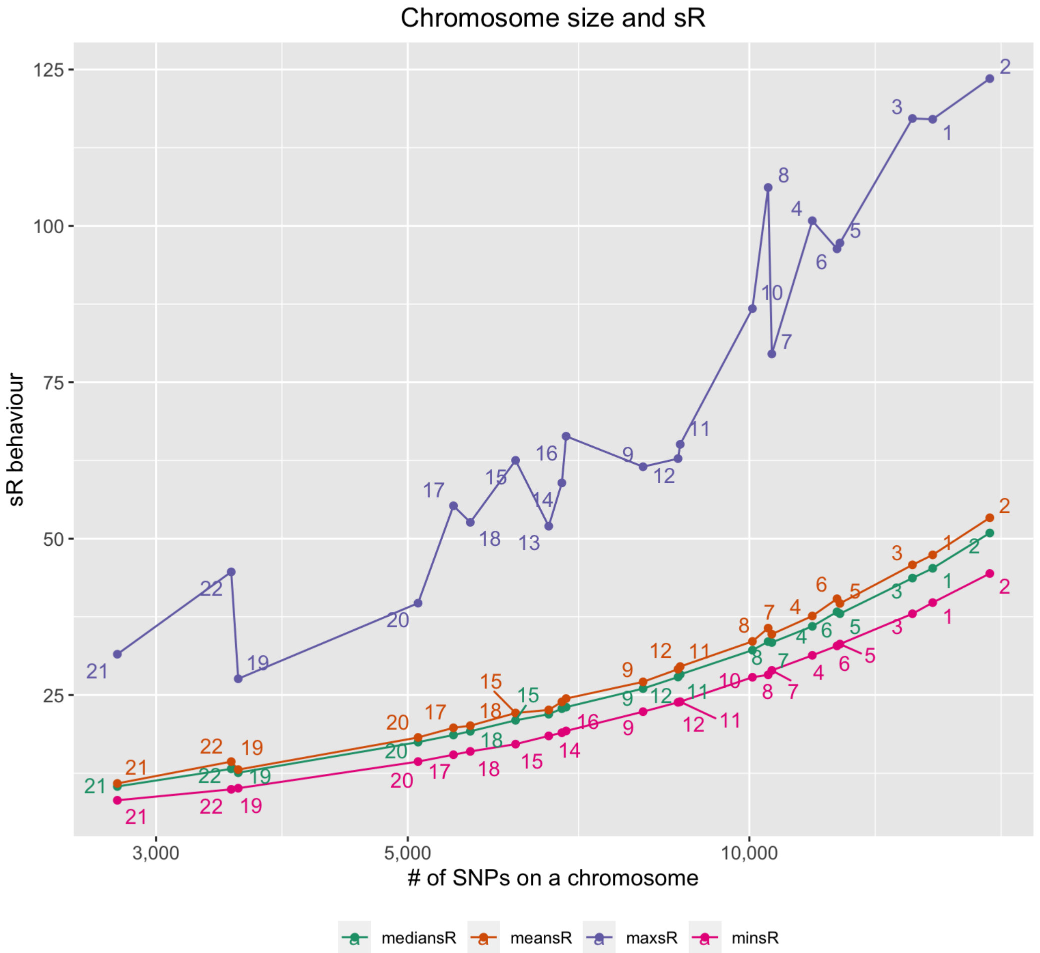

Figure S10: A summary of multi-collinearity as a function of chromosome size for populations in Africa.

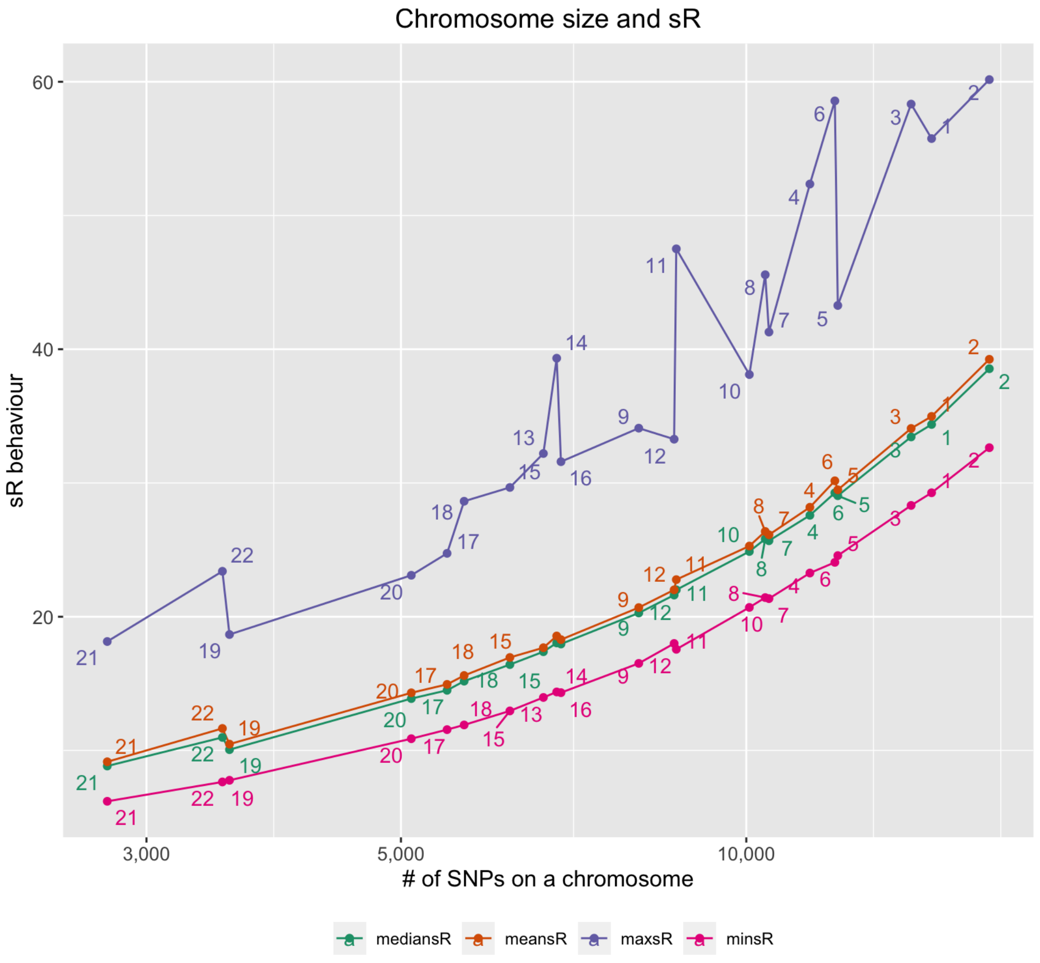

Figure S12: A summary of multi-collinearity as a function of chromosome size for populations in East Asia.

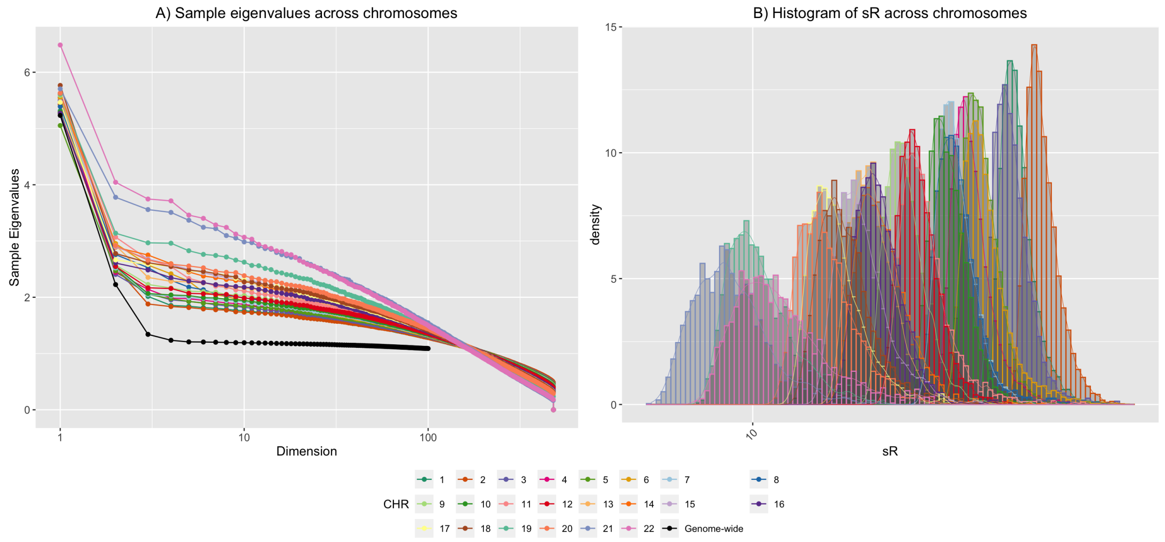

Figure S13: Patterns of multi-collinearity measured by $\{sR_j\}_j$ in East Asia.

References

Lee H Dicker. Variance estimation in high-dimensional linear models. Biometrika, 101(2):269–284, 2014.

Péter Kovács, Tibor Petres, and László Tóth. A new measure of multicollinearity in linear regression models. International Statistical Review, 73(3):405–412, 2005.

Alan Stuart, Steven Arnold, J Keith Ord, Anthony O'Hagan, and Jonathan Forster. Kendall's advanced theory of statistics. London, Wiley, 1994.

Bradley Efron. Large-scale inference: empirical Bayes methods for estimation, testing, and prediction. Cambridge, Cambridge University Press, 2010.
